# Isomer-dependent catalytic pyrolysis mechanism of the lignin model compounds catechol, resorcinol and hydroquinone†

**DOI:** 10.1039/d1sc00654a

**Published:** 2021-02-18

**Authors:** Zeyou Pan, Allen Puente-Urbina, Andras Bodi, Jeroen A. van Bokhoven, Patrick Hemberger

**Affiliations:** Laboratory for Synchrotron Radiation and Femtochemistry, Paul Scherrer Institute 5232 Villigen Switzerland patrick.hemberger@psi.ch; Institute for Chemical and Bioengineering, Department of Chemistry and Applied Biosciences, ETH Zurich 8093 Zurich Switzerland; Laboratory for Catalysis and Sustainable Chemistry, Paul Scherrer Institute 5232 Villigen Switzerland

## Abstract

The catalytic pyrolysis mechanism of the initial lignin depolymerization products will help us develop biomass valorization strategies. How does isomerism influence reactivity, product formation, selectivities, and side reactions? By using imaging photoelectron photoion coincidence (iPEPICO) spectroscopy with synchrotron radiation, we reveal initial, short-lived reactive intermediates driving benzenediol catalytic pyrolysis over H-ZSM-5 catalyst. The detailed reaction mechanism unveils new pathways leading to the most important products and intermediates. Thanks to the two vicinal hydroxyl groups, catechol (*o*-benzenediol) is readily dehydrated to form fulvenone, a reactive ketene intermediate, and exhibits the highest reactivity. Fulvenone is hydrogenated on the catalyst surface to phenol or is decarbonylated to produce cyclopentadiene. Hydroquinone (*p*-benzenediol) mostly dehydrogenates to produce *p*-benzoquinone. Resorcinol, *m*-benzenediol, is the most stable isomer, because dehydration and dehydrogenation both involve biradicals owing to the *meta* position of the hydroxyl groups and are unfavorable. The three isomers may also interconvert in a minor reaction channel, which yields small amounts of cyclopentadiene and phenol *via* dehydroxylation and decarbonylation. We propose a generalized reaction mechanism for benzenediols in lignin catalytic pyrolysis and provide detailed mechanistic insights on how isomerism influences conversion and product formation. The mechanism accounts for processes ranging from decomposition reactions to molecular growth by initial polycyclic aromatic hydrocarbon (PAH) formation steps to yield, *e.g.*, naphthalene. The latter involves a Diels–Alder dimerization of cyclopentadiene, isomerization, and dehydrogenation.

## Abstract 2

Detection of reactive intermediates with synchrotron radiation and photoelectron photoion coincidence methods reveals new mechanistic insights into lignin catalytic pyrolysis. Here we focus on how the isomerism changes the conversion and product formation.

## Graphical abstract

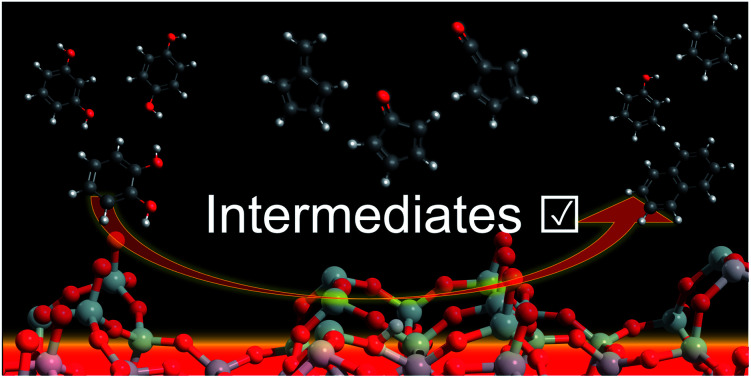

## Introduction

Lignin, one of the main biomass components, can be converted to fuels and fine chemicals by pyrolysis.^1–3^ Due to its varied functionalization and complex structure, even state-of-the-art approaches suffer from limited selectivity and controllability.^4–7^ To optimize conversion and improve selectivity in a targeted way, it is necessary to understand the chemistry of early lignin pyrolysis products. Studies on representative lignin building blocks, such as guaiacol, syringol and eugenol, can provide detailed insight into the (catalytic) pyrolysis mechanism.^8–12^ Li *et al.* used electron paramagnetic resonance (EPR) spectroscopy to show that guaiacol pyrolysis is a radical-driven process and catechol is a favored product due to the presence of mobile hydrogen atoms.^8^ Verma and Kishore carried out density functional theory (DFT) calculations and showed that the lowest-energy unimolecular decomposition pathway of eugenol yields guaiacol.^9^ Detailed mechanistic studies exist for benzaldehyde,^13^ phenol,^14^ dimethoxybenzenes^15^ and larger model systems, such as 4-phenoxyphenol and 2-methoxy-phenoxybenzene.^16^

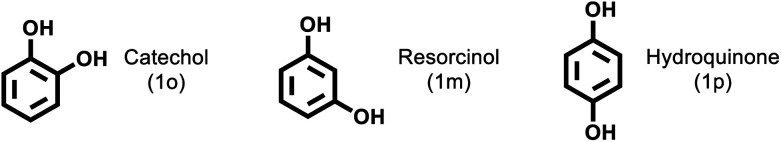



Benzenediols, and especially catechol (1,2-benzenediol, **1o**), represent basic structural units in the lignin network, are major intermediates or products in lignin thermal decomposition, and stand out because of their reactivity, thanks to the presence of two hydroxyl groups. A detailed mechanistic description of benzenediol pyrolysis would be of great value to describe related derivatives and construct models to predict their behavior in lignin decomposition. Thus, it is of fundamental interest to elucidate the pyrolysis mechanism fully by focusing on the role of isomerism and the change in the mechanism in the presence of a zeolite catalyst.

Yang *et al.* investigated catechol, resorcinol, and hydroquinone (*o*-, *m*- and *p*-benzenediol) pyrolysis in a two-stage tubular reactor, analyzed the products by online gas chromatography and reported isomer-dependent product distributions. The *p*-benzoquinone product was only observed for hydroquinone (*p*-benzenediol), while resorcinol (*m*-benzenediol) produced significantly more CO_2_ and C_5_ hydrocarbons than the other two isomers.^17^ Based on computations, a biradical reaction pathway was proposed.^18^ By using *in situ* photoelectron photoion coincidence (PEPICO) methods, we found compelling spectroscopic evidence (*vide infra*) that the resorcinol decomposition mechanism is dominated by a retro-Diels–Alder reaction to produce CO_2_ and C_5_H_6_ isomers (R1) instead or it leads to two highly reactive ketenes species (R1).^18,19^ Furthermore, the three benzenediols share the same reaction to form cyclopenta-2,4-dien-1-ol (C_5_H_6_O) and 2,4-cyclopentadiene-1-one (C_5_H_4_O), initiated by CO loss in a phenolic keto–enol tautomerism pathway (see R2 for an overview).
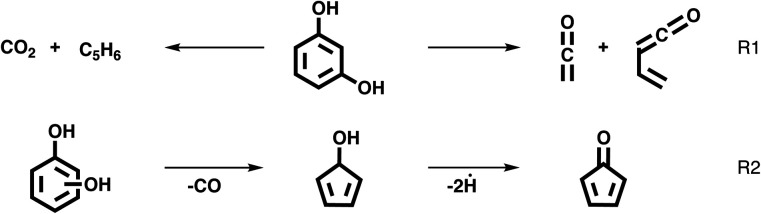


The pyrolysis mechanism of catechol was investigated by Ormond *et al.* using matrix isolation infrared spectroscopy and iPEPICO detection.^20^ Fulvenone, a highly reactive intermediate, was produced only in catechol, because of the vicinal hydroxyl groups opening up the dehydration reaction R3.
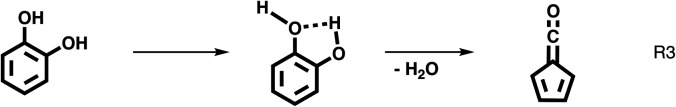


They proposed that the phenolic keto–enol tautomerism pathway of catechol, *i.e.*, carboxyl group formation, followed by CO and H losses, may also apply to resorcinol and hydroquinone (see R2),^20^ as confirmed later for resorcinol.^19^ Based on these considerations, we can clearly expect (i) different reaction pathways for the three benzenediol isomers which can only be revealed by (ii) *in situ* and *operando* detection methods.

How does the reactivity, the intermediates, and the products change in the presence of a catalyst? How does the catalyst influence the benzenediol pyrolysis depending on the arene substitution pattern? Yang *et al.* pyrolyzed catechol with and without H-ZSM-5 at 550–950 °C and reported that the catalyst lowers the reaction temperature and increases the amount of aromatic hydrocarbons produced *via* deoxygenation. However, they did not discuss mechanistic insights in detail.^21^ We have investigated the catalytic pyrolysis of guaiacol and found catechol as a demethylation product. It dehydrates to the highly reactive fulvenone (see R3), which either forms cyclopentadiene *via* decarbonylation and hydrogenation, or phenol *via* radical pathways. Thus, the fulvenone ketene was identified as the central intermediate.^22^ Ketenes have recently been in the spotlight because of their crucial role in numerous catalytic processes.^23^ Although most studies observe catechol in lignin catalytic pyrolysis, isomer-dependent reaction mechanisms remain elusive, probably owing to the limited time resolution and/or isomer specificity of conventional analysis methods.^24–27^ Imaging photoelectron photoion coincidence (iPEPICO) spectroscopy is a multiplexed, sensitive and isomer-selective technique^28^ that allows us to identify reactive intermediates and derive a reaction mechanism in complex and reactive systems ranging from combustion and pyrolysis to catalysis.^19,20,22,29–31^ This is achieved by utilizing photoion mass-selected threshold photoelectron spectra (ms-TPES) for isomer-selective detection of the key intermediates. Reactive intermediates and products are softly ionized using vacuum ultraviolet (VUV) synchrotron radiation, producing photoions and photoelectrons that are detected in delayed coincidence. In addition to mass spectra, the photoelectron spectrum associated to individual *m*/*z* peaks can be plotted for chemical analysis. This represents a powerful tool to detect and assign reactive and short-lived intermediates in benzenediol catalytic pyrolysis isomer-specifically, as detailed in the ESI.†^31^

This study aims to understand the chemistry of H-ZSM-5-catalyzed pyrolysis of the benzenediols catechol, resorcinol and hydroquinone. Specifically, we want to elucidate how isomerism influences the pyrolytic conversion, reactive intermediates, and the reaction mechanism to yield aromatics with and without a zeolite catalyst. These insights will lay the foundation to study even more complex lignin model compounds and to control selectivity and conversion in biomass valorization.

## Results and discussion

### Gas chromatography–mass spectrometry

**Fig. 1:**
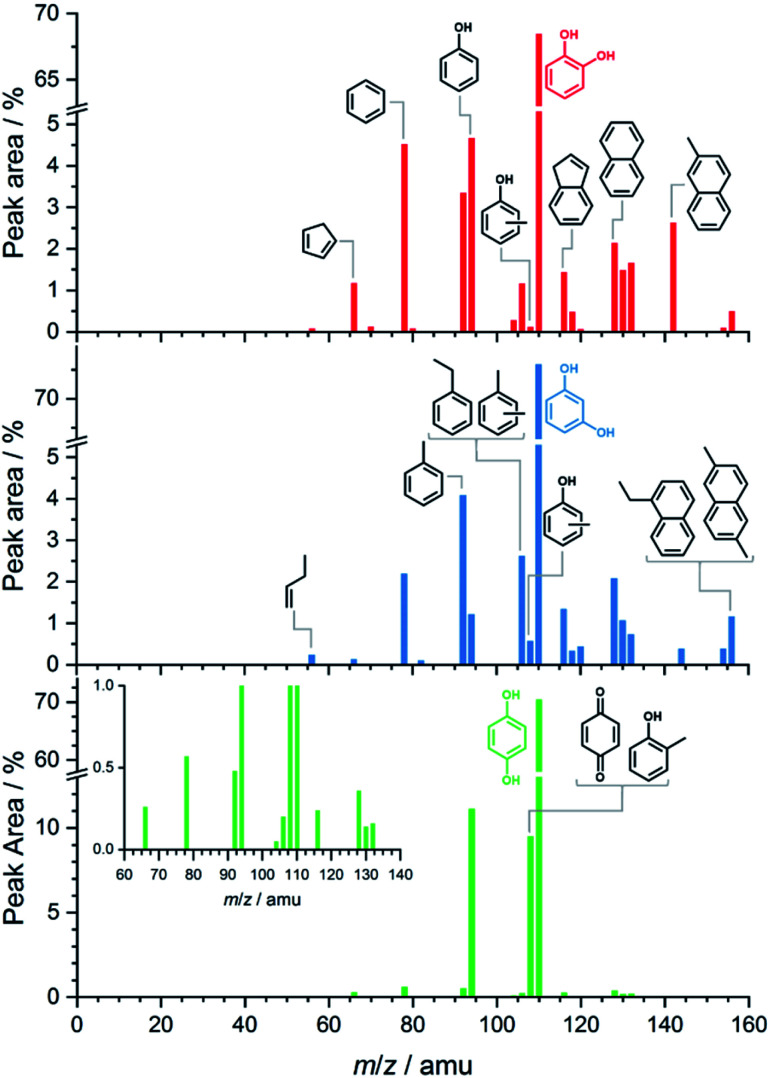
py-GC/MS results of catechol (red sticks, top), resorcinol (blue sticks, center) and hydroquinone (green sticks, bottom) obtained at 530 °C. Besides small molecular and oxygen-containing products, monocyclic and polycyclic aromatic hydrocarbons, *i.e.*, coke precursors, are observed.

Fig. 1 shows the benzenediol product assignment after catalytic pyrolysis in a batch-type reactor by gas chromatography–mass spectrometry (py-GC/MS) based on the NIST08 mass spectrum library. The detailed assignment is shown in the ESI† in Tables S1–S3.† Based on the integral peak intensities in the ion chromatograms (Fig. S1†), catechol has the highest reactivity and largest conversion. Besides phenol, cyclopentadiene and benzene, polycyclic aromatic hydrocarbons (PAHs) such as naphthalene, indene and their alkyl-substituted derivatives dominate the spectra. These are the products of secondary reactions, including decomposition, methylation, isomerization processes and molecular growth, due to the high concentration and long residence time of the reactants, intermediates, and products in the reactor. Reactive species, such as fulvenone (*m*/*z* 92), 2-cyclopenten-1-one (*m*/*z* 82) and ketene (*m*/*z* 42), evade detection because of their short lifetime and the GC/MS detection limits. While resorcinol shows similar products as catechol, it does so at a much lower conversion (see Fig. S1†). Hydroquinone has a higher selectivity towards phenol and *p*-benzoquinone with fewer and less intense contributions of larger aromatics.

Overall, py-GC/MS gives a detailed picture of the final pyrolysis products, such as phenol and PAHs, but does not allow for insights into the initial stages that govern the whole reaction mechanism. Thus, we investigated the pyrolysis chemistry with and without catalyst using our *in situ* PEPICO setup.

### Photoelectron photoion coincidence spectroscopy

#### Catalytic benzenediol decomposition

A continuous injection molecular beam experiment, including a sample container and quartz reactor (see Scheme S1†), was used to extend the lifetime of the reactive intermediates and investigate the initial reaction steps. The solid sample is vaporized in the container and mixes with the carrier gas. It reacts on the catalyst surface in the quartz reactor, packed with glass wool, at *ca.* 100 mbar total pressure. Products and intermediates exiting the catalytic pyrolysis reactor expand into high vacuum, form a molecular beam, are skimmed and then ionized by VUV synchrotron radiation from the Swiss Light Source (Paul Scherrer Institute).^32^ The ions and electrons are accelerated in a constant electric field and detected by velocity map imaging detectors to measure photoionization mass spectra. Peaks in the mass spectra were isomer-selectively identified by their photoion mass-selected threshold photoelectron spectra (ms-TPES) combined with adiabatic ionization energy (AIE) calculations^33^ and Franck–Condon (FC) simulations,^34^ or reference spectra.

**Fig. 2:**
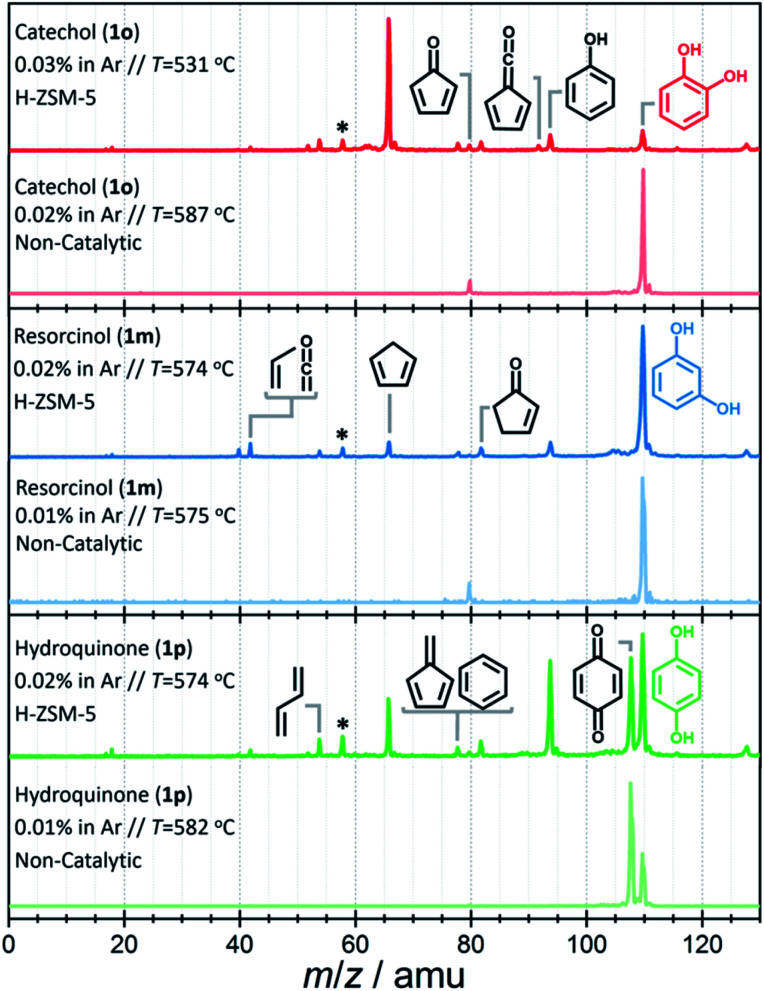
Time-of-flight mass spectra recorded at *hν* = 10.5 eV upon non-catalytic (light-colored) *vs.* catalytic (dark-colored) fast pyrolysis of catechol (red trace, top), resorcinol (blue trace, center) and hydroquinone (green trace, bottom). Catalyst: H-ZSM-5. *Acetone impurity in the chamber background.

First, catalytic and non-catalytic pyrolysis can be compared with the help of the spectra shown in Fig. 2 and S2.† The mass spectra of catechol, resorcinol, and hydroquinone are relatively clean in non-catalytic pyrolysis (Fig. 2, light-colored traces), and only few products are observed at temperatures as high as 580 °C. While we observe *m*/*z* 80 in catechol and resorcinol, which can be assigned to cyclopentadienone (c-C_5_H_4_=O, see R2), we see exclusively *p*-benzoquinone (*m*/*z* 108) in hydroquinone. These findings agree with unimolecular decomposition products reported earlier.^19,20^

Upon adding H-ZSM-5 catalyst, the conversion increases significantly, especially for catechol (see Fig. 2, dark-colored traces), in agreement with the observations of Yang *et al.*,^21^ while the other two isomers show notably increased reactivity only at higher temperatures. A more detailed temperature comparison on catalytic and non-catalytic pyrolysis for catechol, resorcinol and hydroquinone is presented in Fig. S2.† The differences in conversion among the isomers become even more pronounced at low temperatures and if the three isomers are directly compared at the same conditions, as shown in Fig. S3.†

**Fig. 3:**
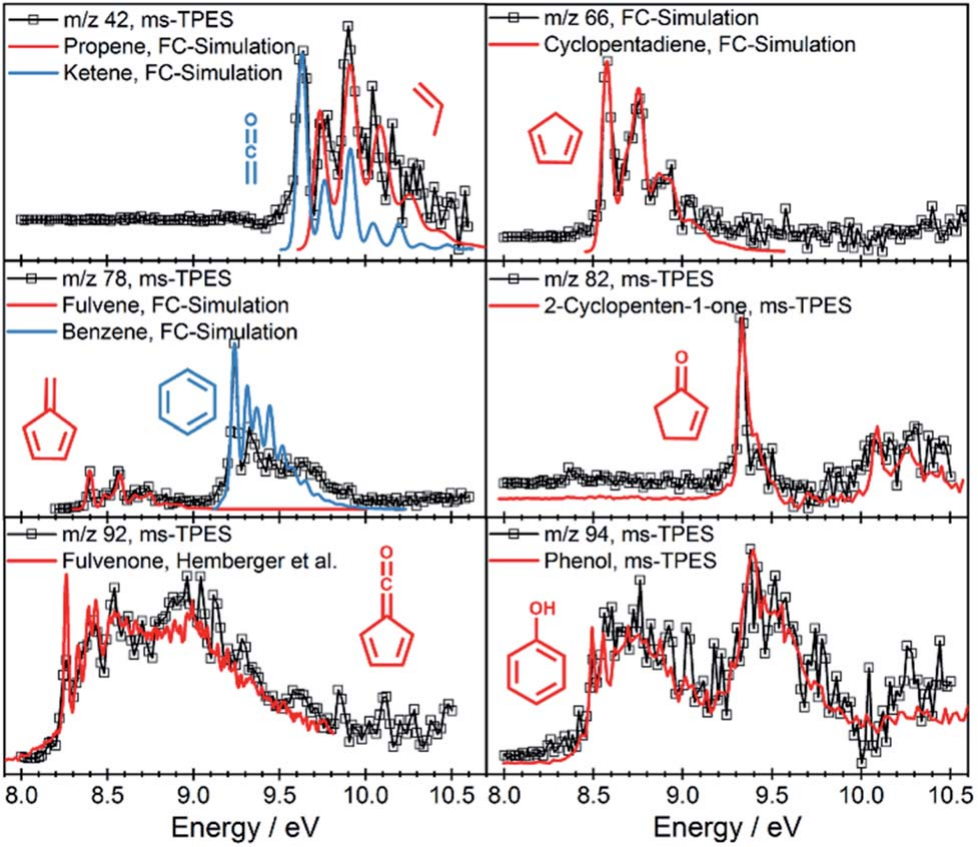
Representative ms-TPE spectra of products along with FC simulations or reference spectra.^35^

The intermediates and products in the mass spectra were analyzed *via* ms-TPE spectroscopy and the most important assignments are depicted in Fig. 3. A direct comparison of the ms-TPES between the benzenediols is given in Fig. S4 and Table S4 in the ESI.† Catechol (*m*/*z* 110, Fig. 2 dark red trace) has a relatively high selectivity towards *m*/*z* 66, which is assigned to cyclopentadiene (see Fig. 3). Furthermore, we assigned fulvene and benzene (both *m*/*z* 78), 2,4-cyclopentadiene-1-one (*m*/*z* 80), 2-cyclopenten-1-one (*m*/*z* 82), phenol (*m*/*z* 94), fulvenone (*m*/*z* 92) and naphthalene (*m*/*z* 128), as intermediates and products of the catechol catalytic pyrolysis.

The second most reactive benzenediol is hydroquinone (*m*/*z* 110, Fig. 2 dark green trace). The main contributors to the mass spectrum are the pyrolysis products cyclopentadiene (*m*/*z* 66), phenol (*m*/*z* 94) and *p*-benzoquinone (*m*/*z* 108), which are observed at higher temperatures compared to catechol. The product distribution of resorcinol (*m*/*z* 110, Fig. 2 dark blue trace) resembles that of catechol but exhibits the lowest conversion among the three benzenediols in the complete temperature range studied. While cyclopentadiene was only observed in the gas-phase pyrolysis of resorcinol,^19,20^ it is seen in all three benzenediols over H-ZSM-5, which is clearly a distinguishing feature of catalytic pyrolysis. This raises the possibility that there is benzenediol isomerization on the catalyst surface. Although most of the assigned intermediates and products are shared among the three benzenediols, we only found fulvenone in high concentrations in the catechol experiments (see *m*/*z* 92 ms-TPES in Fig. S4†). In the 8.5–10.0 eV photon energy range, the fulvenone TPES is dominated by transitions into the strongly coupled ground and first electronically excited state of the cation.^35^ The *m*/*z* 92 ms-TPES barely rises above the background in the resorcinol and hydroquinone experiments, which precludes assignment (Fig. S4†). In such cases, we can rely on the photoionization (PI) spectrum, which includes all ionization events leading to the *m*/*z* channel of interest, not only the threshold ones. The *m*/*z* 92 PI spectra in resorcinol and hydroquinone catalytic pyrolysis are virtually identical (Fig. S5†) and agree perfectly with the fulvenone reference spectrum below 8.7 eV.

Starting from 8.9 eV, the spectrum rises more steeply than the fulvenone reference, which indicates a significant toluene contribution. This proves that the three benzenediols catalytically interconvert, because only the ortho isomer, catechol, can dehydrate to yield fulvenone, thanks to the vicinal hydroxyl groups (see R3).^20^ In the absence of a catalyst, these isomerizations are observed neither in previous pyrolysis experiments^19,20^ nor herein (see Fig. 2). Benzenediol interconversion is further verified by the detection of *p*-benzoquinone (*m*/*z* 108), the dehydrogenation product of hydroquinone, also in the resorcinol and catechol experiments (Fig. S4†). In contrast to fulvenone, *p*-benzoquinone is a stable compound that could have been detected by py-GC/MS of resorcinol and catechol, but it was not, probably because of the detection limit of this technique. Therefore, benzenediol catalytic isomerization is a catalytic side channel compared with the decomposition processes.

**Scheme 1:**
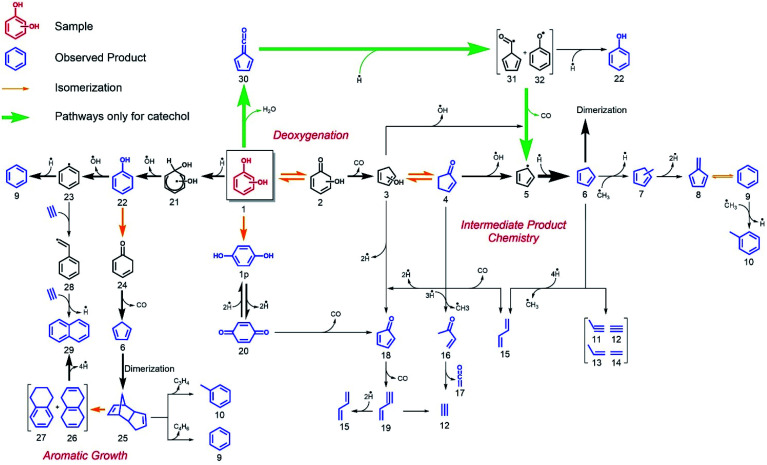
H-ZSM-5-catalyzed pyrolysis reaction mechanism of the three benzenediol isomers. Thick arrows represent the dominant reaction pathways, leading to the most abundant species.

The catechol mechanism can be described as follows (Scheme 1): fulvenone was shown to be the key species in the formation of cyclopentadiene (*m*/*z* 66) and phenol (*m*/*z* 94) from catechol in the catalytic fast pyrolysis of guaiacol.^22^ In brief, catechol (**1o**) is dehydrated in an acid-catalyzed reaction on the catalyst surface, yielding fulvenone (**30**, Scheme 1), which is subsequently hydrogenated to form C_6_H_5_O radicals (**31** and **32**), which decarbonylate and hydrogenate to produce cyclopentadiene (**6**) or hydrogenate to phenol (**22**).^22^ A second benzenediol deoxygenation reaction channel is initiated by decarbonylation to access five-membered ring species, such as hydroxyl cyclopentadienes (**3**) and 2-cyclopenten-1-one (**4**, see R2). This reaction is also observed in non-catalytic pyrolysis but at comparably higher temperatures and was explored by quantum chemical calculations in resorcinol.^19,20^ In addition, benzenediols (**1**) may dehydroxylate to yield phenol (**22**) and water in a Brønsted acid catalyzed reaction. Furthermore, hydroquinone (**1p**) can dehydrogenate to form *p*-benzoquinone (**20**), as summarized in Scheme 1. It is worth noting that benzenediol dehydrogenation reaction is always endothermic, as seen by the CBS-QB3-calculated reaction enthalpies for R4–R6:
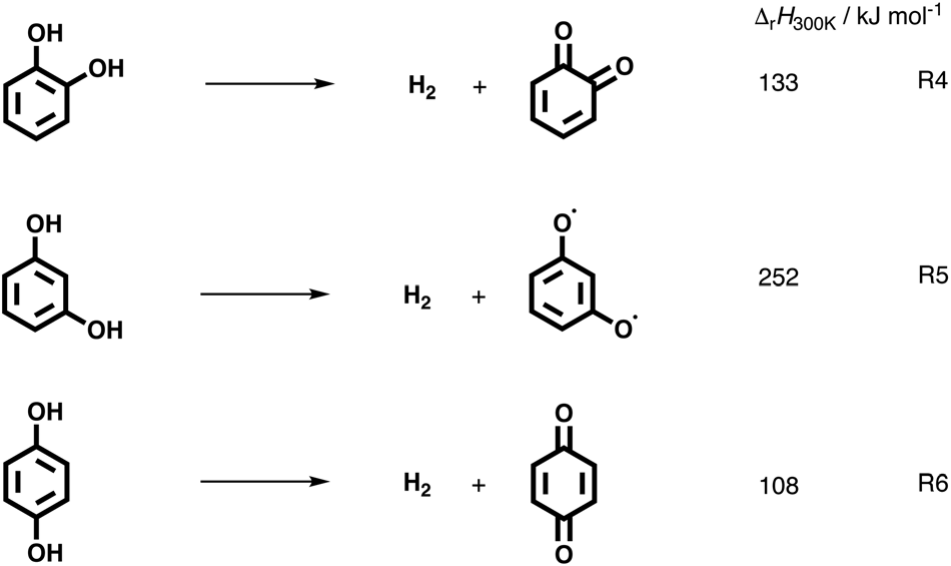


While *o*- and *p*-benzoquinone (C_6_H_4_O_2_) formation are similarly endothermic, the formation of the biradical *meta*-isomer requires twice as much energy and is, thus, less likely to occur during the catalytic pyrolysis of resorcinol. *o*-Benzoquinone may indeed be thermodynamically accessible, but not detected, which can be explained by its high reactivity towards decarbonylation.^15^

These insights into the early reaction pathways are valuable. However, we found them to be lacking to fully understand the mechanism leading to the catalytic pyrolysis products observed in GC^21^ or in py-GC/MS experiments herein. Especially, the dehydroxylation and PAH formation mechanisms seem elusive, which are nevertheless crucial in lignin conversion. We therefore decided to dig deeper and reveal the chemistry of the prompt pyrolysis products more in detail.

#### Intermediate product chemistry

**Fig. 4:**
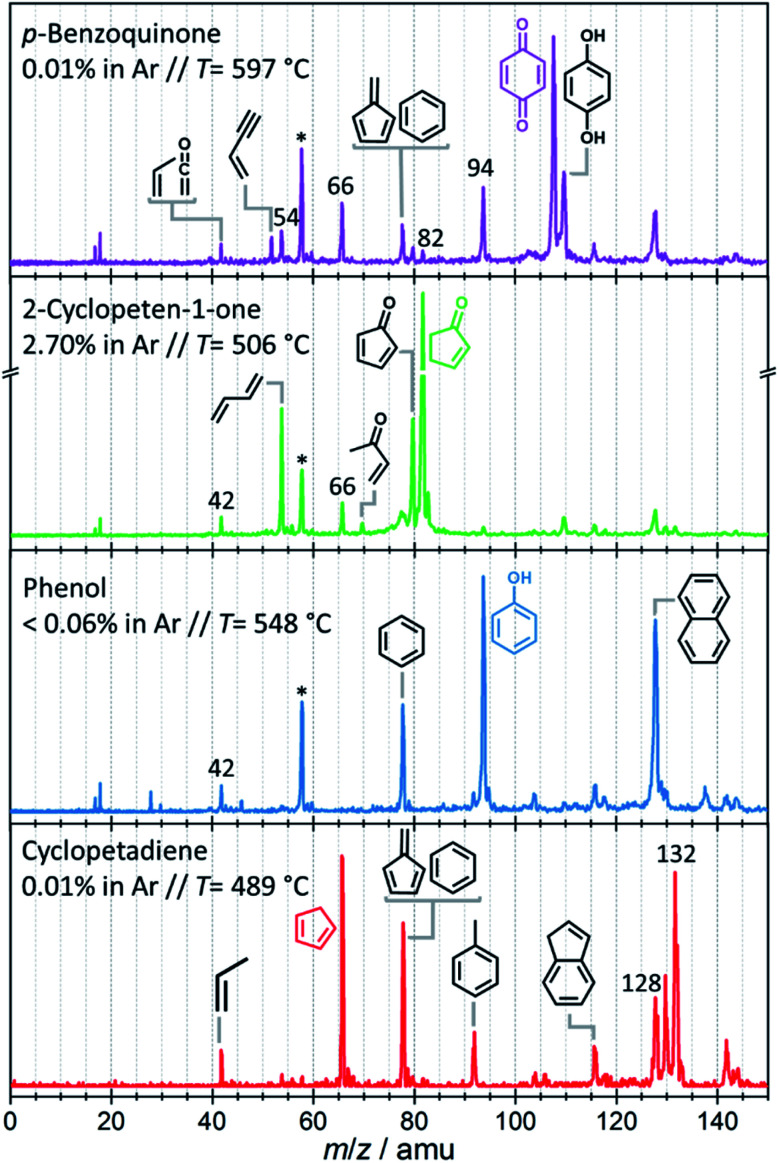
Time-of-flight mass spectra at *hν* = 10.5 eV obtained upon catalytic pyrolysis of *p*-benzoquinone (purple trace), 2-cyclopenten-1-one (green trace), phenol (blue trace) and cyclopentadiene (red trace) over H-ZSM-5. *Acetone impurity in chamber background.

While condensation reactions are crucial in PAH-formation (see below), decomposition processes yield progressively smaller products and only abundantly available species on the catalysis surface may participate, such as hydrogen or, at most, methyl radicals.^22^ Thus, one can shed more light on the catalytic pyrolysis mechanism of benzenediols by investigating the sequential catalytic pyrolysis reactions of the prompt pyrolysis products individually and selectively. The mass spectra for the catalytic pyrolysis of *p*-benzoquinone (*m*/z 108), 2-cyclopenten-1-one (*m*/*z* 82), phenol (*m*/*z* 94), and cyclopentadiene (*m*/*z* 66) are shown in Fig. 4. As before, the intermediates and products were assigned based on their ionization energy and threshold photoelectron spectrum (Fig. 3, S6–S9 and Table S4†). In the *p*-benzoquinone experiment (Fig. 4, purple trace), we identified phenol (*m*/*z* 94), 2-cyclopenten-1-one (*m*/*z* 82), 2,4-cyclopentadiene-1-one (*m*/*z* 80), fulvene (*m*/*z* 78), benzene (*m*/*z* 78), cyclopentadiene (*m*/*z* 66), 1,3-butadiene (*m*/*z* 54), ketene (*m*/*z* 42) and propene (*m*/*z* 42) as pyrolysis products. The product distribution agrees quite well with that of hydroquinone (Fig. 2 dark green trace). This suggests that hydroquinone (**1p**) is easily formed by hydrogenation of *p*-benzoquinone (**20**) (see Scheme 1), due to the abundance of hydrogen on the H-ZSM-5 catalyst. As *p*-benzoquinone is an important contributor to the hydroquinone spectra, as well, we can establish that hydroquinone and *p*-benzoquinone both readily desorb from the catalyst surface and that there is an equilibrium between them thanks to fast interconversion. This is not in contradiction with the strongly endothermic dehydrogenation enthalpy for R6, because the hydrogen atoms are more strongly bound on the surface than in a hydrogen molecule, which decreases the effective dehydrogenation energy significantly. In addition to the products formed *via* hydroquinone, the direct decarbonylation of *p*-benzoquinone (C_6_H_4_O_2_) yields 2,4-cyclopentadiene-1-one (**18**, C_5_H_4_O), which can decarbonylate (see Scheme 1) again to produce 1-buten-3-yne (**19**, C_4_H_4_) as shown in R7:
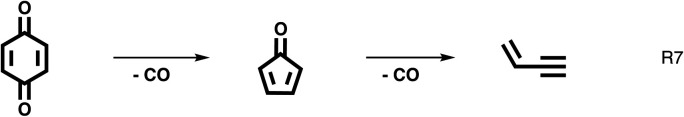


These reactions were also experimentally observed and computationally confirmed by Robichaud *et al.* during the pyrolysis of *p*-dimethoxybenzene, which intermediately yields *p*-benzoquinone.^15^ 1-Buten-3-yne (**19**) is prone to hydrogenation to yield 1,3-butadiene (**15**) on the catalyst surface (Scheme 1). Besides, it may also yield acetylene (**12**) and ethylene at elevated temperatures. PAHs (*e.g.* naphthalene and indene) are also observed at elevated temperatures, analogous to our py-GC/MS results. Their formation mechanism is discussed below.

The green trace in Fig. 4 shows the mass spectrum of 2-cyclopenten-1-one catalytic pyrolysis. We assign the mass spectral peaks to 2,4-cyclopentadiene-1-one (*m*/*z* 80), 2-butenone (*m*/*z* 70), cyclopentadiene (*m*/*z* 66), 1,3-butadiene (*m*/*z* 54), ketene (*m*/*z* 42), and propene (*m*/*z* 42) with the help of ms-TPE spectra (Fig. S7†). 2-Cyclopenten-1-one (**4**) can dehydrogenate on the catalyst surface to yield 2,4-cyclopentadiene-1-one (**18**) or demethylate *via* ring opening to form 2-butenone (**16**) (see Scheme 1) after hydrogenation over H-ZSM-5. The latter can decompose further to yield ketene (**17**) along with acetylene (**12**). The loose methyl groups on the catalyst surface can readily methylate the products (**6** → **7**), similar to what was observed in guaiacol catalytic pyrolysis.^22^ Decarbonylation of **4** results in 1,3-butadiene (**15**). Furthermore, cyclopentadiene (**6**) was identified in small quantities, which could tentatively be produced by a sequence of Brønsted acid catalyzed hydrogenation and dehydroxylation reactions of 2-cyclopenten-1-one (**4** → **6**).

#### Probing deoxygenation channels

Phenol catalytic pyrolysis shows a much simpler picture and reveals the dehydroxylation reaction mechanism of phenolics. Benzene (*m*/*z* 78) and naphthalene (*m*/*z* 128) are the major products, while ethylene (*m*/*z* 28), propene (*m*/*z* 42), and ketene (*m*/*z* 42) are observed only in small quantities (Fig. 4 blue trace). The dehydroxylation of phenol on the catalyst surface (Scheme 1, **22** → **23** → **9**) yields benzene together with only trace amounts of fulvene (*m*/*z* 78 ms-TPES in Fig. S8†). This is a crucial finding as it points to two formation channels of C_6_H_6_ (*vide infra*). In addition, phenol (**22**) can isomerize to 2,4-cyclohexadienone (**24**) *via* phenolic keto-enol tautomerization, which decarbonylates to cyclopentadiene (**6**, Scheme 1) as observed also during non-catalytic pyrolysis and confirmed *via* quantum chemical calculations by Scheer *et al.* (R8):^36^
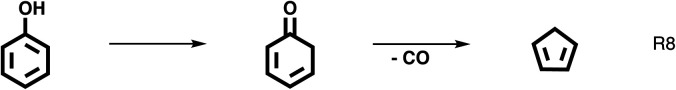


Cyclopentadiene (**6**) is only observed in very low concentrations (see Fig. S10†) during phenol catalytic pyrolysis due to its high reactivity (*vide infra*).

While these measurements have filled in the gaps in the mechanism, they have not revealed details of aromatic growth and PAH formation, a crucial step in coking and catalyst deactivation. Indeed, the ms-TPES of *m*/*z* 128 shows contributions from 1-buten-3-ynylbenzene and/or 3-buten-1-ynylbenzene (see Fig. S9†) beside naphthalene, as verified by FC simulations and points towards complex PAH chemistry. For instance, phenyl radicals (**23**) could recombine with acetylene to naphthalene (**29**) in hydrogen abstraction–acetylene addition (HACA)^37–40^ (Scheme 1, **23** → **28** → **29**), but neither the **28** intermediate nor ethynylbenzene were observed. **23**, a non-resonantly stabilized radical, may require higher activation energy, and the pathway (**23** → **28** → **29**) may only play a minor role. In addition, we found traces of *m*/*z* 152 (Fig. S10†), which can tentatively be assigned as ethynyl naphthalene or acenaphthylene.^40,41^ Thus, a more detailed investigation of the early PAHs and coke intermediates is necessary to unlock their formation mechanism.

### Probing aromatic growth

**Scheme 2:**
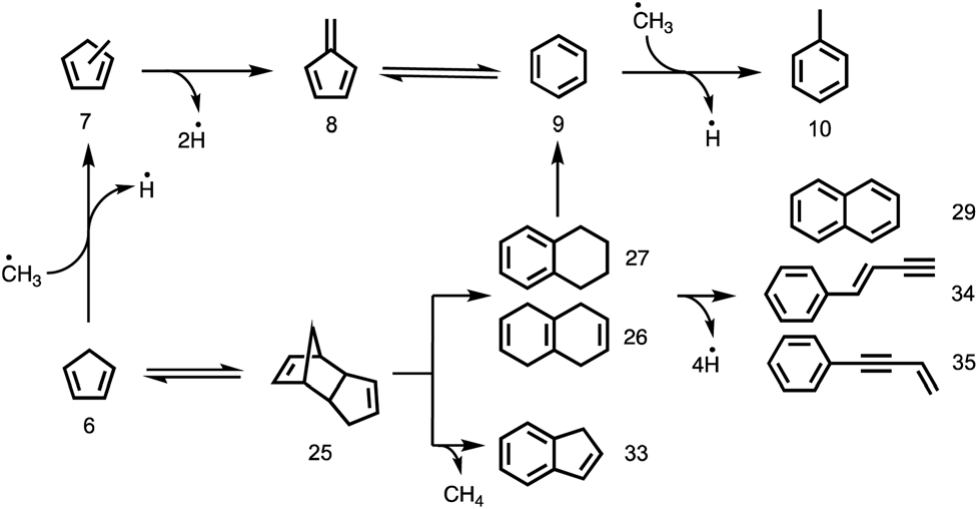
Formation of polycyclic aromatic compounds *via* dimerization of cyclopentadiene and subsequent dehydrogenation or C–C bond cleavage.

The catalytic pyrolysis of cyclopentadiene, presented in Fig. 4 (red trace), offers valuable insights into PAH and early coke formation.^42^ Interestingly, cyclopentadiene completely disappeared from the spectrum at the highest temperature, 582 °C (Fig. S11† left). This also explains why it is only present in trace amounts in phenol catalytic pyrolysis (Fig. 4 blue trace). The propene (*m*/*z* 42) peak in cyclopentadiene catalytic pyrolysis confirms that cyclopentadiene is a source of C_3_ species by ring opening to produce propene and acetylene/ethylene.^14^ C_3_ moieties, such as allyl (C_3_H_5_) and propargyl (C_3_H_3_) radicals, are benzene precursors par excellence, as was recently found in propane oxyhalogenation over CrPO_4_.^43^ However, the low propene signal points towards other PAH formation channels in the phenol experiments.^43^ It is well known that cyclopentadiene (**6**) can dimerize and dehydrogenate to naphthalene (**29**), as shown in Schemes 1 and 2.^44,45^ Besides decomposition and dimerization, cyclopentadiene (**6**) may also grow by formal addition of CH_3_ units, yielding fulvene (**8**), benzene (**9**) and toluene (**10**). The peak at *m*/*z* 80 could be assigned to a combination of three methyl-cyclopentadiene isomers (Fig. S9†), which implies methylation on the catalyst and likely contributes to fulvene formation *via* dehydrogenation (**6** → **7** → **8**).^22^

Interestingly, large contributions (Fig. 4 red trace) of naphthalene (**29**), 1-buten-3-ynylbenzene, 3-buten-1-ynylbenzene to *m*/*z* 128 and of tetrahydronaphthalenes (C_10_H_12_, **26**, **27**) and dicyclopentadiene (C_10_H_12_, **25**) to *m*/*z* 132 are found in our ms-TPES analysis (see Fig. S9†). Assuming that their ionization cross sections are similar, it is eye-catching that the bicyclic C_10_H_12_ isomers have a comparable if not higher abundance in the mass spectrum than the monocyclic toluene and benzene/fulvene (Fig. 4 red trace). As the C_5_H_6_ dimer was not observed at room temperature and in the absence of the catalyst, it must be formed during the reaction of cyclopentadiene on H-ZSM-5. Thus, it is conceivable that the species above *m*/*z* 66 are produced *via* dimerization of cyclopentadiene, isomerization, and subsequent decomposition to fulvene/benzene and C_4_ (**25** → **9**) species or toluene and C_3_ species **(25** → **10**), respectively.^45^ The latter chemistry is corroborated by the observation of 1-buten-3-ynylbenzene or 3-buten-1-ynylbenzene besides naphthalene (all *m*/*z* 128).

**Fig. 5:**
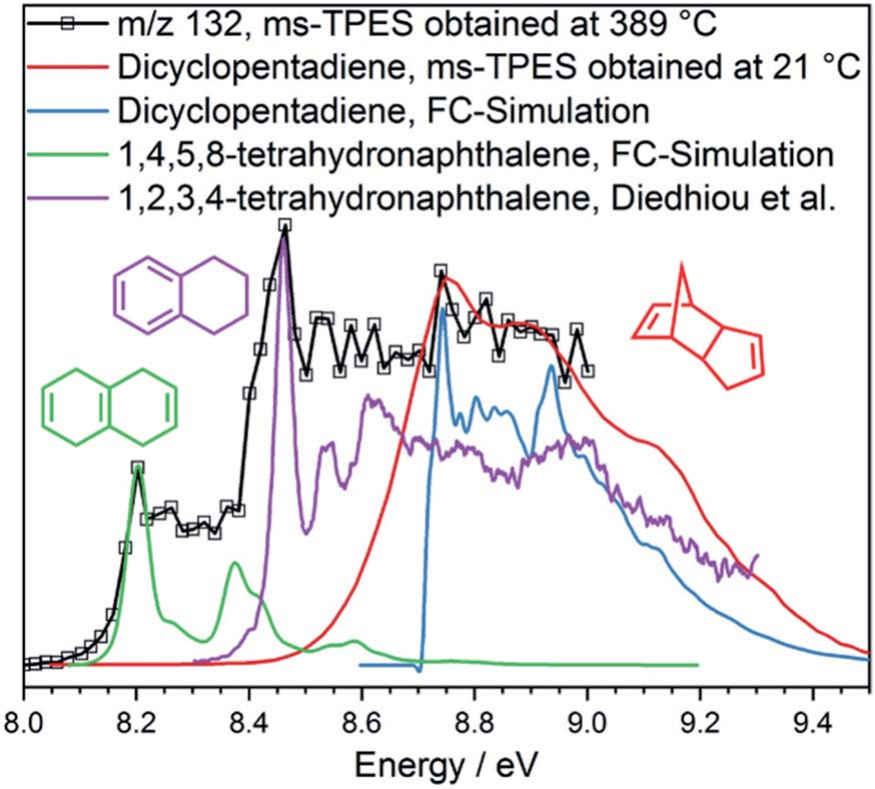
ms-TPE spectra of *m*/*z* 132 in dicyclopentadiene at 21 °C and its catalytic pyrolysis at 389 °C. Isomerization reactions to tetrahydronaphthalenes^47^ are clearly observed.

To verify this hypothesis, we investigated the catalytic pyrolysis of dicyclopentadiene, as shown in Fig. S11† right. The appearance of cyclopentadiene proves that the dimer undergoes a retro-Diels–Alder reaction on the catalyst surface, which is an endothermic process.^46^ The product distribution of dicyclopentadiene is similar to that of cyclopentadiene (Fig. S11† left), and naphthalene (*m*/*z* 128) appeared at elevated temperatures, too. In order to understand the dimerization and formation of naphthalene, we measured the ms-TPES of *m*/*z* 132 during H-ZSM-5-catalyzed pyrolysis of dicyclopentadiene (Fig. 5). We found three isomers of the composition C_10_H_12_ at 389 °C reactor temperature. Two isomers are assigned to 1,2,3,4- and 1,4,5,8-tetrahydronaphthalene (**27**, **26**), while the band at 8.7 eV is attributed to dicyclopentadiene (**25**) in both the *exo* and *endo* forms. This shows that dicyclopentadiene indeed isomerizes first and then continually dehydrogenates *via* formal 2H_2_ loss reactions to yield naphthalene or ring-opens to produce the two buten-ynylbenzenes (**34** and **35**). The possible reaction pathways of dicyclopentadiene (**25**) in catalytic pyrolysis is presented in more detail in Scheme 2 and shows strong evidence that cyclopentadiene is the precursor for the formation of early coke precursors such as naphthalene and indene. Our findings are supported by experiments of Sato *et al.*, who reported that dicyclopentadiene yields aromatics such as benzene, toluene, xylene, indene, naphthalene and coke.^45^ This further evidences that C_5_ intermediates are mainly responsible for the PAH formation in the catalytic pyrolysis of benzenediols and, possibly, larger lignin moieties, as well. These reactions likely contribute to the hydrocarbon pool and thus to coke formation, leading to catalyst deactivation.

### Overview of benzenediol catalytic pyrolysis mechanism

We can now derive a reaction mechanism for the catalytic fast pyrolysis of benzenediols, summarized in Scheme 1. Catechol (**1o**) shows the highest conversion, due to the favorable dehydration of the adjacent hydroxyl groups to form fulvenone (**30**), a short-lived intermediate. Fulvenone is hydrogenated to C_6_H_5_O radicals (**31** and **32**), which are either hydrogenated to yield phenol (**22**) on the catalyst surface or decarbonylated and hydrogenated to form cyclopentadiene (**6**). Especially the latter is observed in large quantities at temperatures as high as 500 °C. Fulvenone (**30**) is also a minor intermediate in the catalytic pyrolysis of resorcinol (**1m**) and hydroquinone (**1p**), due to the minor catalytic isomerization channel of the two isomers to catechol (**1o**), observed only in catalytic benzenediol decomposition.^14,20^ However, benzenediol interconversion is a relatively slow process on H-ZSM-5 and does not contribute significantly to the conversion of the less reactive isomers.^48^

All three isomers can be dehydroxylated in the presence of a hydrogen source to yield phenol (**22**), which is one of the major products and only observed in catalytic pyrolysis. Owing to the large hydrogen abundance on H-ZSM-5, phenol (**22**) can be further dehydroxylated catalytically to benzene (**9**) or it decarbonylates to yield cyclopentadiene (**6**). Naphthalene (**29**) can be produced from cyclopentadiene *via* Diels–Alder cycloaddition followed by isomerization to tetrahydronaphthalenes (**26** and **27**) and subsequent dehydrogenation. The third benzenediol decarbonylation pathway, yielding 2-cyclopenten-1-one (**4**), is the main reason for the wide distribution of products. Not only does 2-cyclopenten-1-one (**4**) decompose to olefinic species, such as 1-buten-3-yne (**19**) and 1,3-butadiene (**15**), but it also yields ketene (**17**) and produces cyclopentadiene (**6**) by a second dehydroxylation reaction. Subsequently, the C_5_ species generate C_6_ and C_7_ species either by dimerization of cyclopentadiene (**6**) and subsequent loss of C_4_ or C_3_ species (**25** → **10** and **25** → **9**) or by consecutive methylation steps starting from C_5_ species. The observed methyl cyclopentadienes (**7**) dehydrogenate to fulvene (**8**), which is a benzene (**9**) precursor. Due to the presence of C_2_ and C_3_ species, other recombination reactions are also conceivable, in which cyclopentadiene is ultimately responsible for molecular growth to produce indene (**25** → **33**) or naphthalene (**25** → **26**/**27** → **29**) and coke at later stages of the reaction, as depicted in Scheme 2.

## Conclusions

Catechol, resorcinol, and hydroquinone have isomer-dependent reaction products in non-catalytic gas-phase pyrolysis. While 2-cyclopenten-1-one is a common product for the three benzenediols, ethenone or fulvenone ketenes are only observed in *meta*- and *ortho*-benzenediol, respectively. In catalytic pyrolysis, the reaction temperature could be significantly decreased, and the products are similar. Catechol has the highest reactivity because of the vicinal hydroxyl groups, which promote dehydration and raise the selectivity towards fulvenone production. The detection of these highly reactive species can only be achieved utilizing sensitive, multiplexed, and universal detection tools like PEPICO with VUV synchrotron radiation. Fulvenone represents a shortcut from catechol to cyclopentadiene and phenol formation and explains the higher conversion for catechol. Resorcinol and hydroquinone, on the other hand, exhibit lower conversion but can still produce cyclopentadiene *via* the 2-cyclopenten-1-one intermediate, which can tautomerize to hydroxyl cyclopentadiene. In addition, we found a well-defined reaction pathway to polycyclic aromatic hydrocarbons, such as naphthalene and indene, formed *via* Diels–Alder addition of cyclopentadiene and subsequent dehydrogenation. Our results show that there are several reaction pathways to cyclopentadiene, which is a PAH and coke precursor and is thus responsible for the deactivation of the catalyst. The catalytic pyrolysis mechanism is dominated by deoxygenation and aromatic growth and intermediate product chemistry.

Benzenediols, especially catechol, are common structural moieties in lignin networks. Understanding their reaction mechanism provides the necessary insight to optimize the catalytic pyrolysis process in general. We have shown that cyclopentadiene is one of the major initial catalytic pyrolysis products responsible for the catalyst deactivation, which should be taken into consideration when optimizing the catalyst and process parameters to suppress catalyst deactivation. Our mechanism may also be generalized to predict the fate of larger lignin moieties and take control of the selectivity. For instance, targeted lignin derivatization to produce catechol while suppressing resorcinol and hydroquinone formation may enhance the conversion and selectivity towards aromatics, such as benzene.

## Conflicts of interest

There are no conflicts to declare.

## Supplementary Material

SC-012-D1SC00654A-s001

## References

[cit1] Li C., Zhao X., Wang A., Huber G. W., Zhang T. (2015). Chem. Rev..

[cit2] Schutyser W., Renders T., Van den Bosch S., Koelewijn S. F., Beckham G. T., Sels B. F. (2018). Chem. Soc. Rev..

[cit3] Liu C., Chen X., Liu X., Cui C., Zhou Z., Jia L., Qi F. (2021). Angew. Chem., Int. Ed..

[cit4] Azadi P., Inderwildi O. R., Farnood R., King D. A. (2013). Renewable Sustainable Energy Rev..

[cit5] Mu W., Ben H., Ragauskas A., Deng Y. (2013). BioEnergy Res..

[cit6] Rinaldi R., Jastrzebski R., Clough M. T., Ralph J., Kennema M., Bruijnincx P. C. A., Weckhuysen B. M. (2016). Angew. Chem., Int. Ed..

[cit7] Ma Z., Troussard E., van Bokhoven J. A. (2012). Appl. Catal., A.

[cit8] Li G. X., Luo Z. Y., Wang W. B., Cen J. M. (2020). Catalysts.

[cit9] Verma A. M., Kishore N. (2017). RSC Adv..

[cit10] Wang M., Liu C., Xu X. X., Li Q. (2016). Chem. Phys. Lett..

[cit11] Zhou J., Jin W., Shen D. K., Gu S. (2018). J. Anal. Appl. Pyrolysis.

[cit12] Custodis V. B. F., Hemberger P., Ma Z., van Bokhoven J. A. (2014). J. Phys. Chem. B.

[cit13] Vasiliou A. K., Kim J. H., Ormond T. K., Piech K. M., Urness K. N., Scheer A. M., Robichaud D. J., Mukarakate C., Nimlos M. R., Daily J. W., Guan Q., Carstensen H.-H., Ellison G. B. (2013). J. Chem. Phys..

[cit14] Scheer A. M., Mukarakate C., Robichaud D. J., Nimlos M. R., Carstensen H. H., Ellison G. B. (2012). J. Chem. Phys..

[cit15] Robichaud D. J., Scheer A. M., Mukarakate C., Ormond T. K., Buckingham G. T., Ellison G. B., Nimlos M. R. (2014). J. Chem. Phys..

[cit16] Custodis V. B. F., Hemberger P., van Bokhoven J. A. (2017). Chem. - Eur. J..

[cit17] Yang H., Furutani Y., Kudo S., Hayashi J.-i., Norinaga K. (2016). J. Anal. Appl. Pyrolysis.

[cit18] Furutani Y., Kudo S., Hayashi J. I., Norinaga K. (2017). J. Phys. Chem. A.

[cit19] Gerlach M., Bodi A., Hemberger P. (2019). Phys. Chem. Chem. Phys..

[cit20] Ormond T. K., Baraban J. H., Porterfield J. P., Scheer A. M., Hemberger P., Troy T. P., Ahmed M., Nimlos M. R., Robichaud D. J., Daily J. W., Ellison G. B. (2018). J. Phys. Chem. A.

[cit21] Yang H., Norinaga K., Li J., Zhu W., Wang H. (2018). Fuel Process. Technol..

[cit22] Hemberger P., Custodis V. B. F., Bodi A., Gerber T., van Bokhoven J. A. (2017). Nat. Commun..

[cit23] Chowdhury A. D., Gascon J. (2018). Angew. Chem., Int. Ed..

[cit24] Fu Z. W., Shen Q. R., Yao C. L., Li R., Wu Y. L. (2020). Chemistryselect.

[cit25] Liguori L., Barth T. (2011). J. Anal. Appl. Pyrolysis.

[cit26] Mondal A. K., Qin C. R., Ragauskas A. J., Ni Y. H., Huang F. (2020). Ind. Crop. Prod..

[cit27] Wilson A. N., Dutta A., Black B. A., Mukarakate C., Magrini K., Schaidle J. A., Michener W. E., Beckham G. T., Nimlos M. R. (2019). Green Chem..

[cit28] Bodi A., Hemberger P., Osborn D. L., Sztáray B. (2013). J. Phys. Chem. Lett..

[cit29] Hemberger P., van Bokhoven J. A., Perez-Ramirez J., Bodi A. (2020). Catal. Sci. Technol..

[cit30] Paunovic V., Zichittella G., Hemberger P., Bodi A., Perez-Ramirez J. (2019). ACS Catal..

[cit31] Sztáray B., Voronova K., Torma K. G., Covert K. J., Bodi A., Hemberger P., Gerber T., Osborn D. L. (2017). J. Chem. Phys..

[cit32] Johnson M., Bodi A., Schulz L., Gerber T. (2009). Nucl. Instrum. Methods Phys. Res., Sect. A.

[cit33] Frisch M. J., Trucks G. W., Schlegel H. B., Scuseria G. E., Robb M. A., Cheeseman J. R., Scalmani G., Barone V., Mennucci B., Petersson G. A., Nakatsuji H., Caricato M., Li X., Hratchian H. P., Izmaylov A. F., Bloino J., Zheng G., Sonnenberg J. L., Hada M., Ehara M., Toyota K., Fukuda R., Hasegawa J., Ishida M., Nakajima T., Honda Y., Kitao O., Nakai H., Vreven T., Montgomery J. A., Peralta J. E., Ogliaro F., Bearpark M., Heyd J. J., Brothers E., Kudin K. N., Staroverov V. N., Kobayashi R., Normand J., Raghavachari K., Rendell A., Burant J. C., Iyengar S. S., Tomasi J., Cossi M., Rega N., Millam J. M., Klene M., Knox J. E., Cross J. B., Bakken V., Adamo C., Jaramillo J., Gomperts R., Stratmann R. E., Yazyev O., Austin A. J., Cammi R., Pomelli C., Ochterski J. W., Martin R. L., Morokuma K., Zakrzewski V. G., Voth G. A., Salvador P., Dannenberg J. J., Dapprich S., Daniels A. D., Farkas O., Foresman J. B., Ortiz J. V., Cioslowski J. and Fox D. J., Gaussian 16, Revision A.03, Wallingford CT, 2016

[cit34] Mozhayskiy V. A. and Krylov A. I., ezSpectrum, http://iopenshell.usc.edu/downloads

[cit35] Hemberger P., Pan Z., Bodi A., van Bokhoven J. A., Ormond T. K., Ellison G. B., Genossar N., Baraban J. H. (2020). ChemPhysChem.

[cit36] Scheer A. M., Mukarakate C., Robichaud D. J., Nimlos M. R., Carstensen H.-H., Ellison G. B. (2012). J. Chem. Phys..

[cit37] Frenklach M., Wang H. (1991). Proc. Combust. Inst..

[cit38] Janssen J. M., Senser D. W. (1991). Combust. Flame.

[cit39] Wang H., Frenklach M. (1994). J. Phys. Chem..

[cit40] Parker D. S., Kaiser R. I., Troy T. P., Ahmed M. (2014). Angew. Chem., Int. Ed. Engl..

[cit41] Kislov V. V., Islamova N. I., Kolker A. M., Lin S. H., Mebel A. M. (2005). J. Chem. Theory Comput..

[cit42] Moffett R. B. (1952). Org. Synth..

[cit43] Zichittella G., Hemberger P., Holzmeier F., Bodi A., Perez-Ramirez J. (2020). J. Phys. Chem. Lett..

[cit44] Anderson J. R., Chang F., Western R. J. (1989). J. Catal..

[cit45] Sato A., Miyake T., Sekizawa K. (1994). Appl. Catal., A.

[cit46] Xu R., Jocz J. N., Wiest L. K., Sarngadharan S. C., Milina M., Coleman J. S., Iaccino L. L., Pollet P., Sievers C., Liotta C. L. (2019). Ind. Eng. Chem. Res..

[cit47] Diedhiou M., West B. J., Bouwman J., Mayer P. M. (2019). J. Phys. Chem. A.

[cit48] Weigert F. J. (1987). J. Org. Chem..

