# Supplementary material for: Isomer-dependent catalytic pyrolysis mechanism of the lignin model compounds catechol, resorcinol and hydroquinone
Source: Chem Sci. 2021 Feb 18;12(9):3161–9. doi: 10.1039/d1sc00654a (PMC8179379; doi:10.1039/d1sc00654a)
Supplement: SC-012-D1SC00654A-s001 [file SC-012-D1SC00654A-s001.pdf]

Electronic Supplementary Material (ESI) for Chemical Science.
This journal is © The Royal Society of Chemistry 2021

ELECTRONIC SUPPLEMENTARY INFORMATION

Isomer-Dependent Catalytic Pyrolysis Mechanism of the Lignin Model Compounds Catechol, Resorcinol and Hydroquinone

Zeyou Pan[a,b], Allen Puente-Urbina[b], Andras Bodi[a], Jeroen A. van Bokhoven[b,c], Patrick Hemberger[a]*

[a] Zeyou Pan, Andras Bodi and Patrick Hemberger
Laboratory for Synchrotron Radiation and Femtochemistry, Paul Scherrer Institute, 5232 Villigen, Switzerland. E-mail: patrick.hemberger@psi.ch.
[b] Zeyou Pan, Allen Puente-Urbina, Jeroen A. van Bokhoven
Institute for Chemical and Bioengineering, Department of Chemistry and Applied Biosciences, ETH Zurich, 8093 Zurich, Switzerland
[c] Jeroen A. van Bokhoven, Laboratory for Catalysis and Sustainable Chemistry, Paul Scherrer Institute, 5232 Villigen, Switzerland

Catalytic fast pyrolysis with imaging photoelectron photoion coincidence spectroscopy (py-iPEPICO)

Catalytic fast pyrolysis experiments were carried out using the double imaging photoelectron photoion coincidence endstation CRF-PEPICO at the vacuum ultraviolet beamline (VUV) of the Swiss Light Source (SLS) at Paul Scherrer Institute.[1, 2] Synchrotron radiation is collimated and diffracted by a 150 mm^{-1} grating, and focused onto the exit slit located in a gas filter (Ar, Ne and Kr mixture), which suppresses higher harmonic radiation. The VUV radiation photoionizes molecules in the CRF-PEPICO endstation, which consists of a double velocity map imaging detector assembly for electrons and ions, of which the latter one is also optimized for time-of-flight detection.[2]

The configuration of the pyrolysis (py-iPEPICO) setup (Scheme S1) includes digital mass flow controllers, a sample container, a quartz reactor and the CRF-PEPICO detection chamber. The carrier gas Ar was metered by a digital mass flow controller (20 sccm), and it picks up the vapor of the sample in the sample container. The pressure was recorded by a capacitive gauge. Sample (*e.g.*, catechol) was vaporized in the container, of which the temperature was controlled by a water thermostat (Huber Minichiller) to set the sample concentration in the gas phase, based on its vapor pressure.[3, 4] The sample/Ar mixture entered the quartz reactor ($D_{outside}$ = 4 mm, D_{inside} = 2 mm, heated over a length of 26 mm, including a 1 mm nozzle), packed with 9–11 mg catalyst. Glass wool was used to keep the catalyst in place. A cylindrical wire heater ($D_{inside} \approx$ 4 mm) connected to a DC power supply (Voltcraft) was applied to heat the reactor, and the reactor temperature was monitored by a type K thermocouple attached to the outside reactor wall at the midpoint of the heater. The inside temperature of the reactor was calibrated against the outside temperature and the temperatures in the manuscript refer to the actual catalyst temperature. The gas mixture containing the reactants, as well as the intermediates and products, desorbed from the catalyst surface, leaves the reactor and expands into high vacuum to form a molecular beam. The expansion prevents quenching of the reactive intermediates. The central part of the molecular beam is skimmed and travels towards the photoionization region. After photoionization by vacuum ultraviolet light, the ions and electrons are accelerated in a constant electric field and detected by velocity map imaging detectors utilizing position-sensitive delay line anode detectors in a multi-start/multi-stop coincidence scheme.[2, 5] This assembly enables the measurement of photoion mass-selected

threshold photoelectron spectra and photoion mass spectra, which are used for the isomer-selective detection of reactive intermediates and products.

Catalytic fast Pyrolysis (CFP) in py-GC/MS

In order to compare the reactions to ambient conditions, py-GC/MS was applied to pyrolyze the sample over H-ZSM-5. The mixture (H-ZSM-5-to-sample ratio of 1:4) was ground and packed with quartz wool in a quartz batch reactor heated resistively by a platinum coil pyrolyzer (5150, CDS Analytical). The experiments were carried out at 530 °C for 20 s at a heating rate of 20 °C ms^{-1}. After pyrolysis, helium carrier gas transferred the products at 300 °C into a GC/MS system (Agilent 7890A GC and Agilent 5975 MS) equipped with a HP-5MS capillary column. The GC oven was programmed to start at 40 °C for 5 min and heated up to 200 °C (10 °C min^{-1}) and then to 270 °C for 25 min (20 °C min^{-1}). Products were identified according to the NIST08 mass spectrum library.

Samples

Catechol (Sigma-Aldrich, ≥99%), hydroquinone (Fluka, ≥99%), resorcinol (Sigma-Aldrich, ≥99%), *p*-benzoquinone (Sigma-Aldrich, ≥99.5%), 2-cyclopenten-1-one (Sigma-Aldrich, 98%), phenol, and dicyclopentadiene (Sigma-Aldrich, for synthesis) were used as received. 1,3-Cyclopentadiene was synthesized from dicyclopentadiene according to the literature and preserved at −22 °C.[6]

The ammonium ZSM-5 (Si/Al=25) was purchased from Zeolyst International (CBV 5524G). To obtain hydrogen ZSM-5 (H-ZSM-5), the ammonium ZSM-5 was heated for about 4 h using the heating rate of 2 °C min^{-1} then calcined in static air keeping at 550 °C for 6 h.

Computational details

The Gaussian 16 rev. A.03 suite of programs was used to carry out quantum chemical computations.[7] Optimized geometries and vibrational frequencies were utilized in the Franck–Condon (FC) simulations applying density functional theory at the B3LYP/6-31G(d,p), B3LYP/GTBas3 or B3LYP/6-311G++(d,p)level. The simulations were either carried out with Gaussian 16 rev. A.03 or with ezSpectrum.[8] The stick spectra were convoluted with a Gaussian function and compared to the experimental ms-TPES for isomer-specific assignment.[9] The adiabatic ionization energies for intermediates and products as well as the benzenediol dehydrogenation enthalpies were calculated using the G4 composite method.[10]

Table S1. py-GC/MS results of catechol in CFP using H-ZSM-5. The goodness of the peak assignment is also provided (Qual.).

Peak	Area / %	Retention Time / min	Compound	*m*/*z*	Qual.
1	1.17	2.051	1,3-Cyclopentadiene	66	90
2	4.52	2.900	Benzene	78	91
3	3.34	4.904	Toluene	92	95
4	0.21	7.613	Ethylbenzene	106	91
5	0.95	7.827	Xylene	106	97
6	0.28	8.410	Styrene	104	92
7	4.67	10.558	Phenol	94	91-94
8	0.16	11.505	Indane	118	90
9	1.44	11.667	Indene	116	97
10	0.22	12.343	1-Phenyl-1-butene	132	94
11	0.11	12.834	Benzofuran, 2-methyl-	132	96
12	0.24	13.342	1H-Indene, 2,3-dihydro-5-methyl-	132	95
13	0.89	13.515	1H-Indene, 1-methyl-	130	95
14	0.31	13.723	Naphthalene, 1,2,3,4-tetrahydro-	132	96
15	2.14	14.099	Naphthalene	128	97
16	68.46	14.561	Catechol	110	80-97
17	2.63	15.814	Naphthalene, 2-methyl-	142	96
18	0.77	15.987	1H-Inden-1-one, 2,3-dihydro-	132	98
19	0.18	17.165	Naphthalene, 1-ethyl-	156	95
20	0.19	17.315	Naphthalene, 2,6-dimethyl-	156	97
21	1.3	18.765	Dibenzofuran	168	91
22	0.29	19.597	Fluorene	166	93-94
23	0.35	21.907	Phenanthrene	178	96
24	0.59	22.559	2-Dibenzofuranol	184	93-94
25	0.15	22.894	Phenanthrene, 2-methyl-	192	98

Table S2. py-GC/MS results of resorcinol in CFP using H-ZSM-5. The goodness of the peak assignment is also provided (Qual.).

Peak	Area / %	Retention Time / min	Compound	*m*/*z*	Qual.
1	0.23	1.664	1-Butene	56	64
2	0.07	2.022	1,3-Cyclopentadiene	66	72
3	2.18	2.837	benzene	78	91
4	4.07	4.800	Toluene	92	94
5	0.37	7.555	Ethylbenzene	106	91
6	1.78	7.774	Xylene	106	97
7	0.19	10.009	Benzene, 1-ethyl-2-methyl-	120	95
8	0.17	10.495	Phenol	94	94
9	0.24	10.651	Benzene, 1,2,3-trimethyl-	120	92
10	0.26	11.488	Indane	118	91
11	1.33	11.667	Indene	116	97
12	0.3	11.881	Phenol, 2-methyl-	108	97
13	0.26	12.256	Phenol, 3-methyl-	108	96
14	0.3	12.735	Benzofuran, 2-methyl-	132	91-96
15	0.13	13.336	Benzene, 1-ethenyl-4-ethyl-	132	94
16	1	13.509	1H-Indene, 1-methyl-	130	94-95
17	2.07	14.098	Naphthalene	128	89-97
18	0.28	15.161	1H-Indene, 1,3-dimethyl-	144	91-96
19	75.72	15.721	Resorcinol	110	55-95
20	0.51	17.171	Naphthalene, 1-ethyl-	156	96
21	0.64	17.321	Naphthalene, 2,6-dimethyl-	156	98
22	0.37	17.644	Naphthalene, 2-ethenyl-	154	96
23	0.15	18.586	Naphthalene, 2,3,6-trimethyl-	170	93
24	0.7	18.771	2-Naphthalenol	144	64
25	0.46	20.099	Fluorene	166	93
26	0.22	21.473	9H-Fluorene, methyl-	180	91-97
27	0.23	21.895	Phenanthrene	178	94
28	0.21	22.888	Phenanthrene, 2-methyl-	192	95-97

Table S3. py-GC/MS results of hydroquinone in CFP using H-ZSM-5. The goodness of the peak assignment is also provided (Qual.).

**Peak**	**Area / %**	**Retention Time / min**	**Compound**	***m/z***	**Qual.**
1	0.26	2.120	1,3-Cyclopentadiene	66	90
2	0.57	2.900	Benzene	78	90-91
3	0.48	4.904	Toluene	92	95
4	0.15	7.815	Benzene, dimethyl	106	97
5	10.31	9.103	*p*-Benzoquinone	108	97
6	11.1	10.61	Phenol	94	81-94
7	0.24	11.667	Indene	116	97
8	0.16	11.892	Phenol, methyl-	108	91-96
9	0.14	12.828	Benzofuran, 2-methyl-	132	96
10	0.08	13.515	1H-Indene, 1-methyl-	130	95
11	0.36	14.093	Naphthalene	128	97
12	3.06	14.306	Catechol	110	93-96
13	69.72	15.669	Hydroquinone	110	76-91
14	0.64	18.759	Dibenzofuran	168	76
15	0.07	19.441	Dibenzo-p-dioxin	184	95
16	0.25	21.179	Phenol, 4-phenoxy-	186	96
17	0.37	22.444	2-Dibenzofuranol	184	97

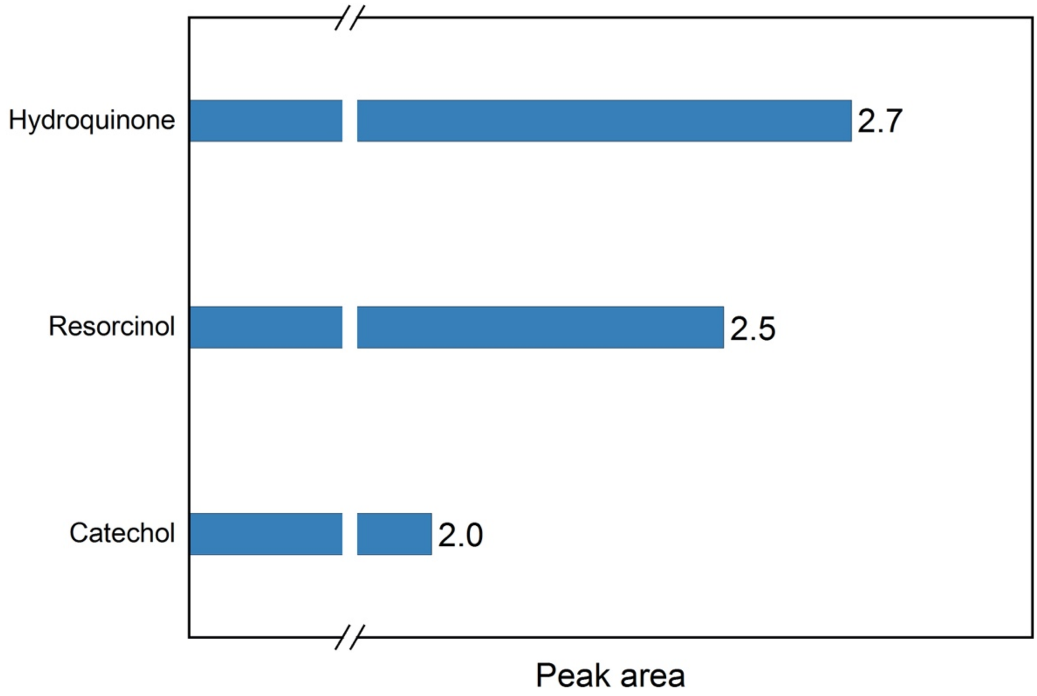

Figure S1. Peak areas of hydroquinone, resorcinol and catechol obtained by py-GC/MS. Based on the same sample amount and assuming similar responses for the three isomers, the figure suggests that catechol has the highest conversion under the same conditions.

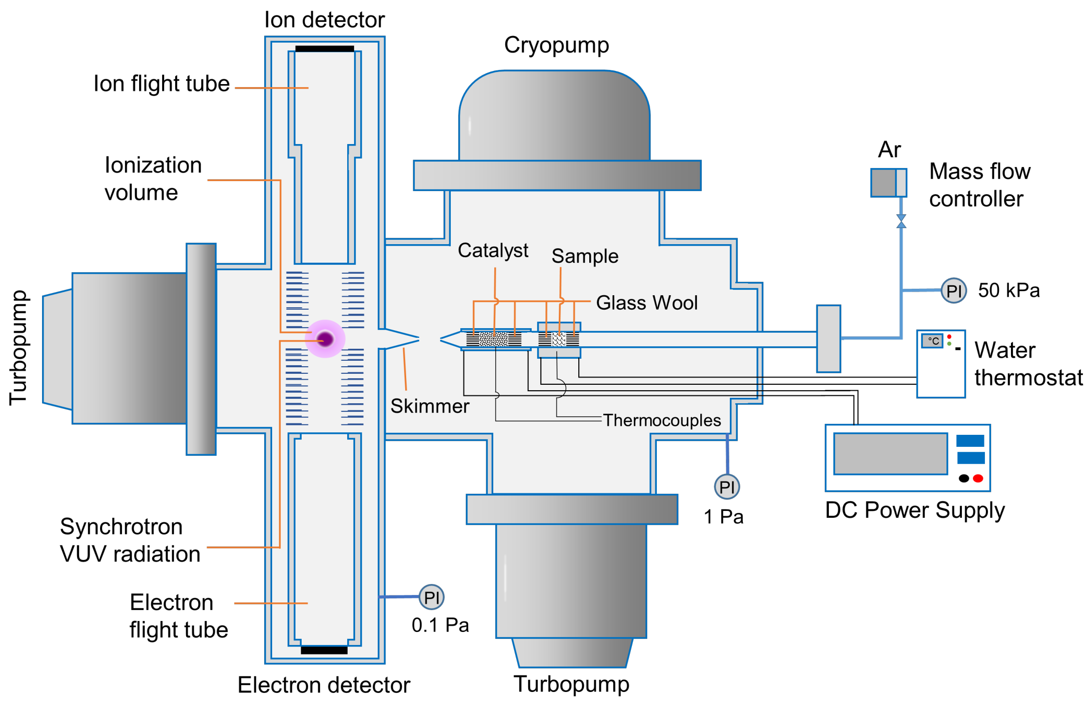

Scheme S1. Schematic diagram of iPEPICO endstation.

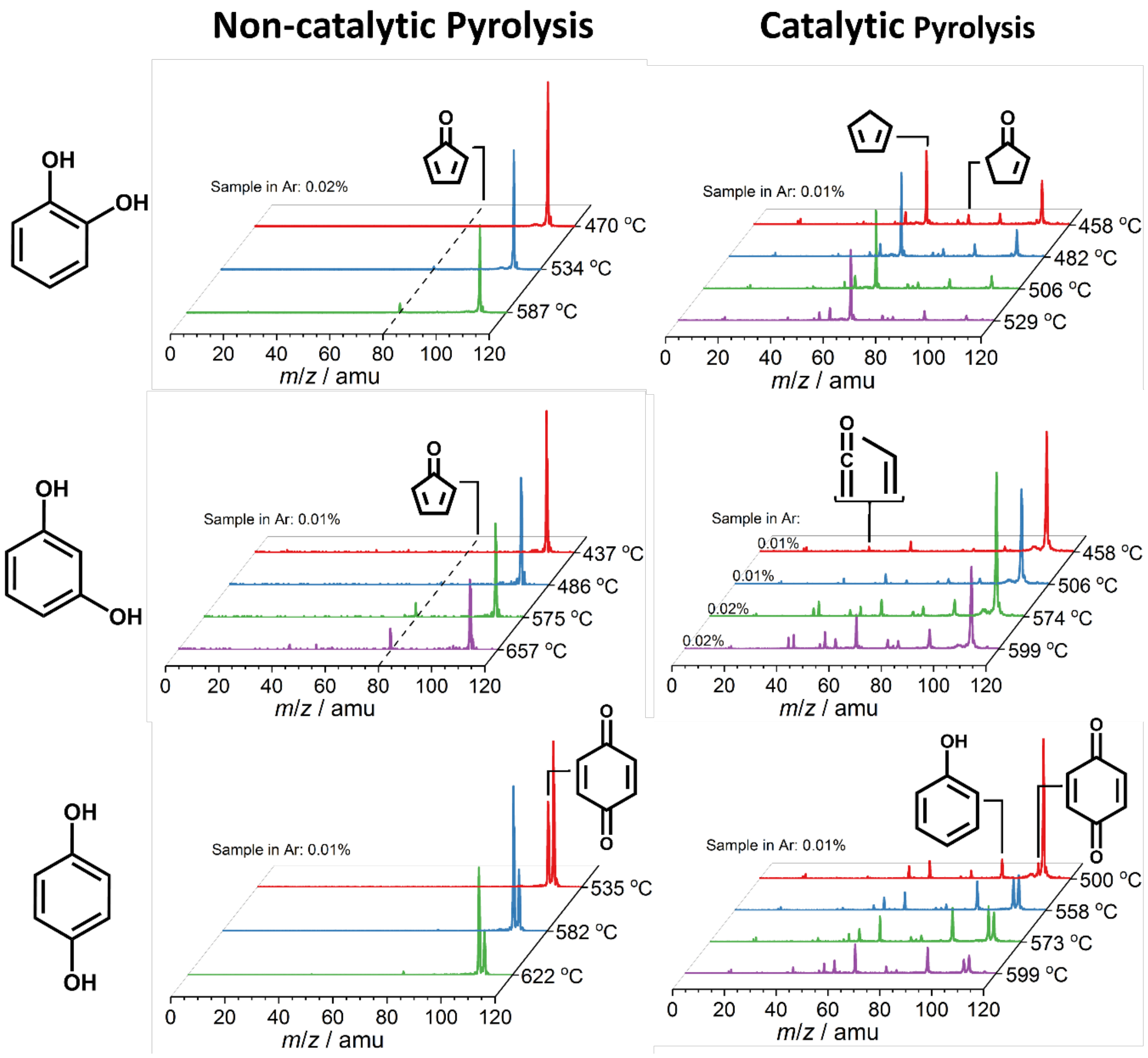

Figure S2. Time-of-flight mass spectra (hv = 10.5 eV) of non-catalytic pyrolysis (left, only quartz glass wool in the reactor) and catalytic pyrolysis (right, H-ZSM-5 and quartz glass wool in the reactor) of benzenediols. In non-catalytic pyrolysis, catechol, resorcinol, and hydroquinone show relatively clean mass spectra with few product peaks even at high temperature. With the addition of H-ZSM-5, similar products are observed for the three isomers at similar conditions, which indicates significant catalyst role in driving conversion and selectivity.

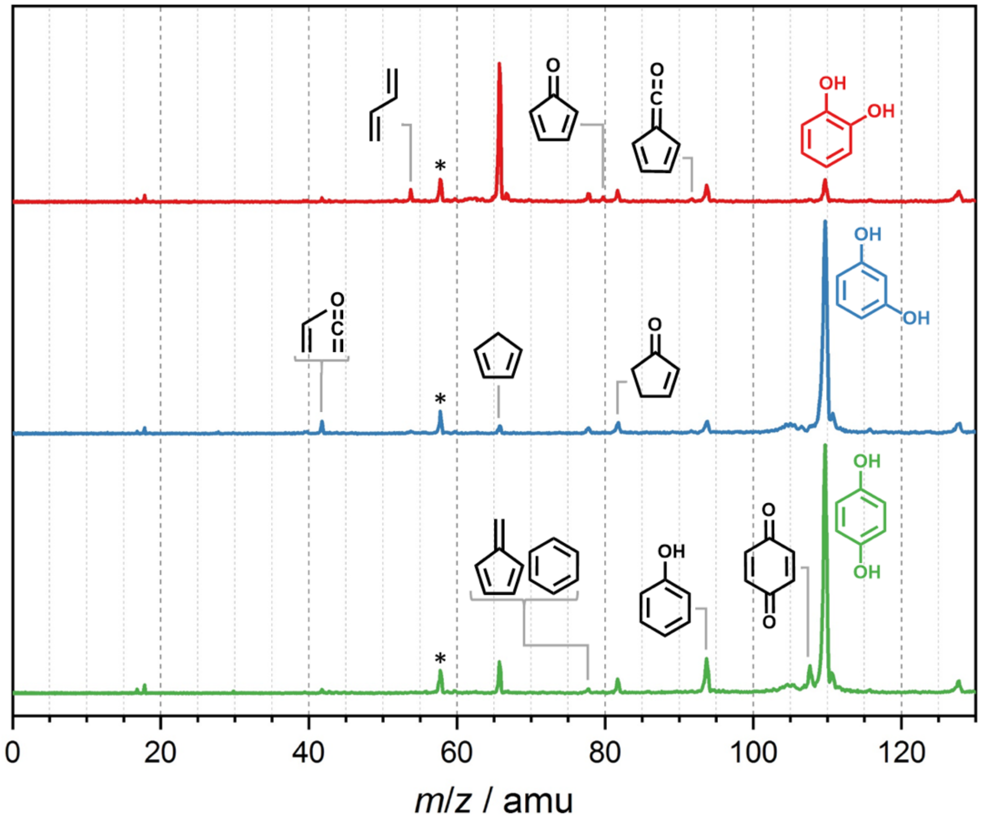

Figure S3. Time-of-flight mass spectra ($h\nu$ = 10.5 eV) obtained upon catalytic pyrolysis of catechol (red trace), resorcinol (blue trace) and hydroquinone (green trace). Conditions: ~506 ºC; H-ZSM-5; ~0.01% benzenediol in Ar. * Acetone impurity in the chamber. At the same conditions, the three isomers show different conversion. Catechol has the highest coversion even at low temperature and cyclopentadiene dominates the products at m/z 66, while resorcinol shows the lowest conversion because it has the highest energy barrier to yield a biradical intermediate (R5 in the main text).

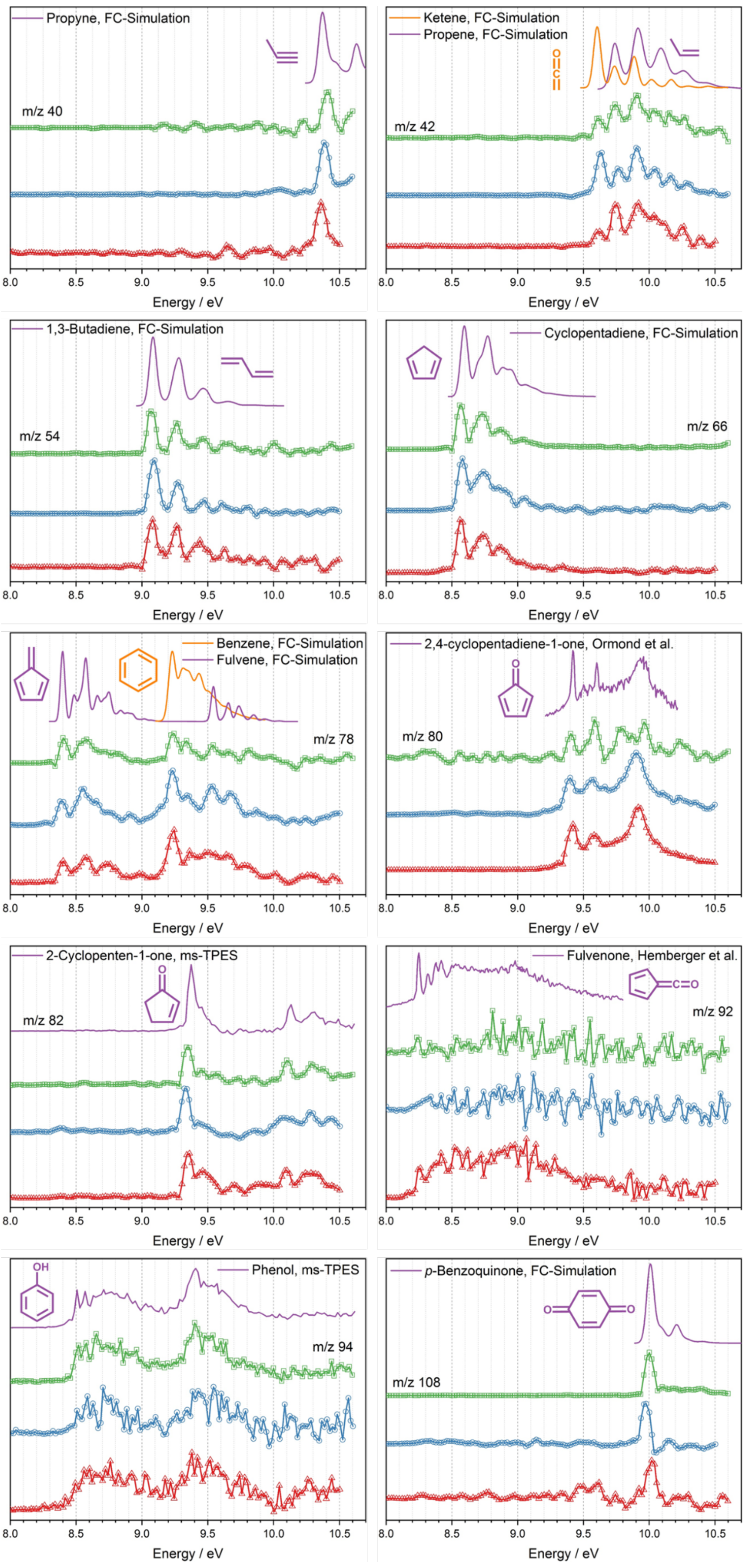

Figure S4. ms-TPE spectra of products along with FC simulations.[11, 12] Red curves are catechol products, blue curves are resorcinol products and green curves are hydroquinone products.

Table S4. Ionization energy (IE) of benzenediol products.

Compound	m/z	IE (eV)		Ref. IE (eV)	Reference
		Calculated[a]	Experimental[b]		
Propyne	40	10.358	10.385	10.37 ± 0.02	13
Propene	42	9.770	9.749	9.7 ± 0.1	
Ketene	42	9.591	9.617	9.61 ± 0.02	14
1-Buten-3-yne	52	9.607	9.596	9.58 ± 0.02	13
1,3-Butadiene	54	9.090	9.080	9.09 ± 0.05	15
Cyclopentadiene	66	8.593	8.569	8.58 ± 0.02	13
Fulvene	78	8.440	8.397	8.36 ± 0.02	
Benzene	78	9.288	9.235	9.27	16
2,4-Cyclopentadiene-1-one	80	9.447	9.407	9.49	17
1-Methylcylopentadiene	80	8.165	-	8.40 ± 0.02	13
2-Methylcylopentadiene	80	8.303	8.281	8.4 (vertical value)	18
5-Methylcylopentadiene	80	8.466	8.45	8.45 ± 0.02	13
2-Cyclopenten-1-one	82	9.578	9.344	9.35	19
Cyclopent-3-en-1-one	82	9.568	-	9.44 ± 0.02	20
Cyclopenta-1,3-dien-2-ol	82	8.073	-	-	-
Cyclopenta-1,3-dien-1-ol	82	7.801	-	-	-
Cyclopenta-2,4-dien-1-ol	82	8.637	-	-	-
Toluene	92	8.836	8.818	8.82	21
Fulvenone	92	8.244	8.271	8.05	22
Phenol	94	8.526	8.575	8.52	23
p-Benzoquinone	108	10.050	9.998	10.01	24
Indene	116	8.181	8.142	8.14 ± 0.01	25
Naphthalene	128	8.148	8.142	8.15	26
1,2,3,4-Tetrahydronaphthalene	132	8.467	8.466	8.44	27
1,4,5,8-Tetrahydronaphthalene	132	8.168	8.204	8.2	28
Dicyclopentadiene	132	8.305	8.70 (vertical value)	8.79 ± 0.05 (vertical value)	29

a: Adiabatic ionization energies (AIE) are calculated at the G4 level of the theory.

b: The energy from the first vibrational peak.

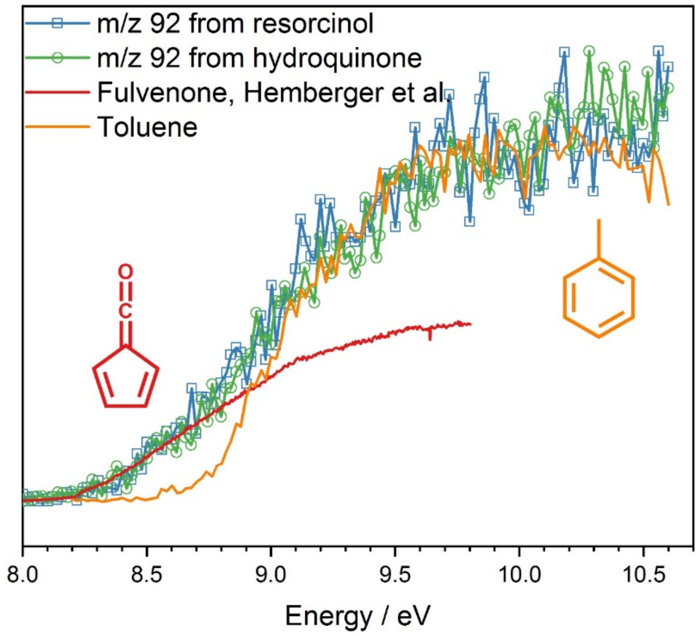

Figure S5. Photoionization (PI) spectrum of *m*/*z* 92 in catalytic pyrolysis as well as fulvenone and toluene spectra.[12] Beginning at *ca.* 8.2 eV, the *m/z* 92 signals increased for resorcinol and hydroquinone, agreeing well with the fulvenone PI spectrum, which suggests that fulvenone was produced in resorcinol and hydroquinone catalytic pyrolysis. This is consistent with *p*-benzoquinone observation in catechol and resorcinol. The benzenediols must isomerize and interconvert for resorcinol and hydroquinone to yield fulvenone, because only the ortho isomer catechol can dehydrate to form fulvenone thanks to the vicinal hydroxyl groups. The PI spectrum rises steeply than the fulvenone reference after *ca.* 8.9 eV, which indicates significant toluene contribution.

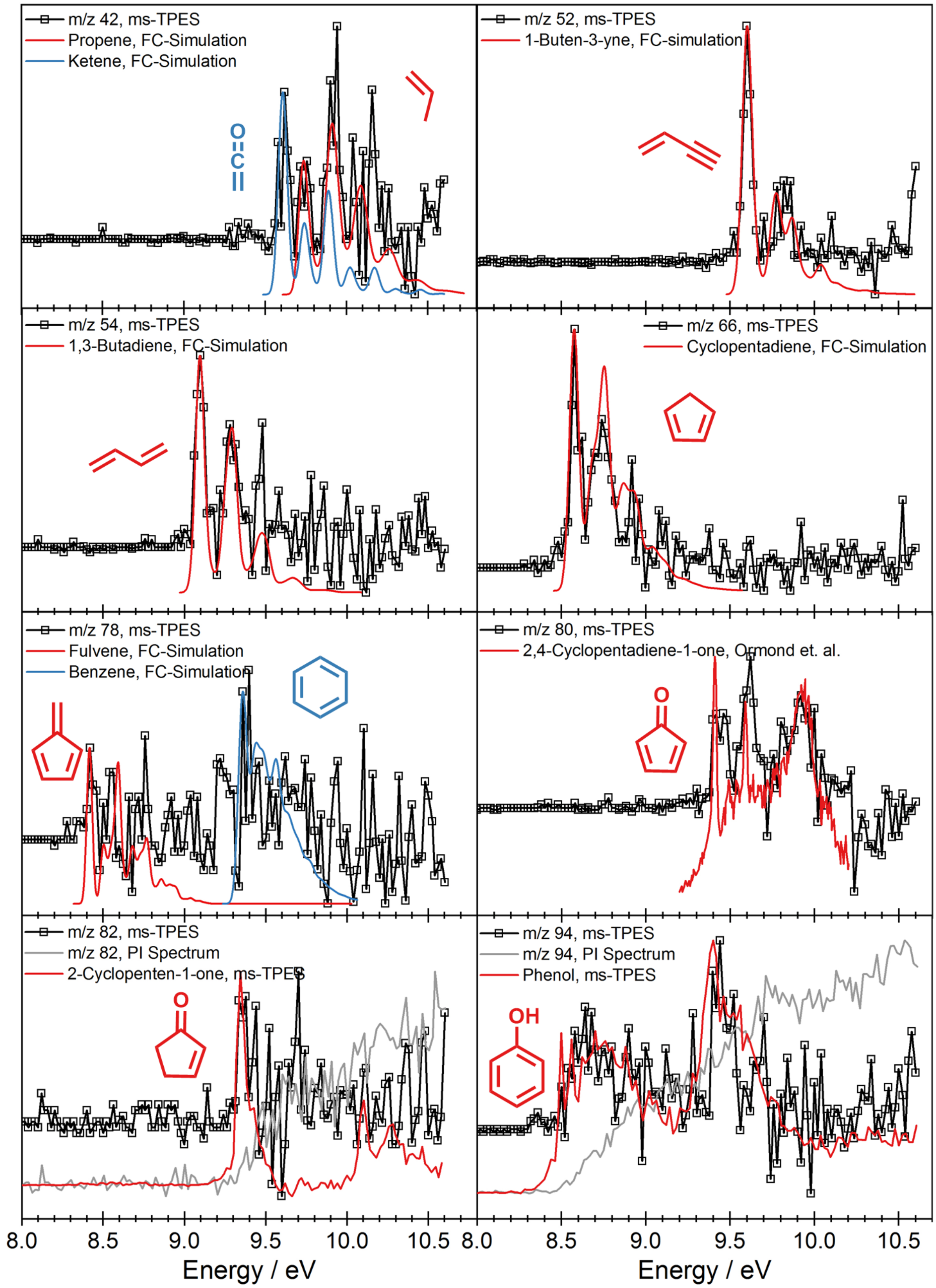

Figure S6. ms-TPE spectra of *p*-benzoquinone products along with FC simulations and PI spectra.[11]

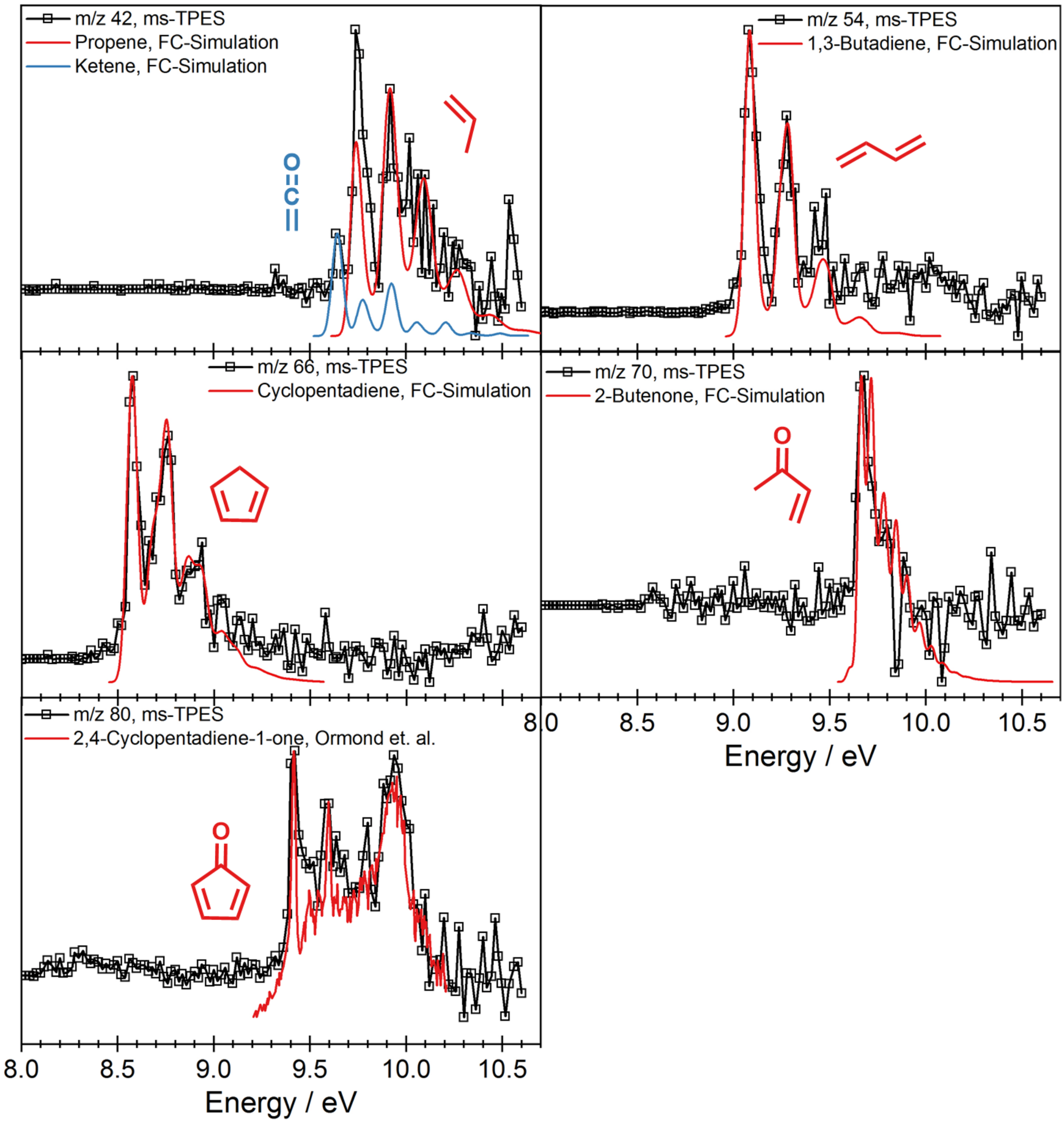

Figure S7. ms-TPE spectra of 2-cyclopenten-1-one products along with FC simulations or reference spectra.[11]

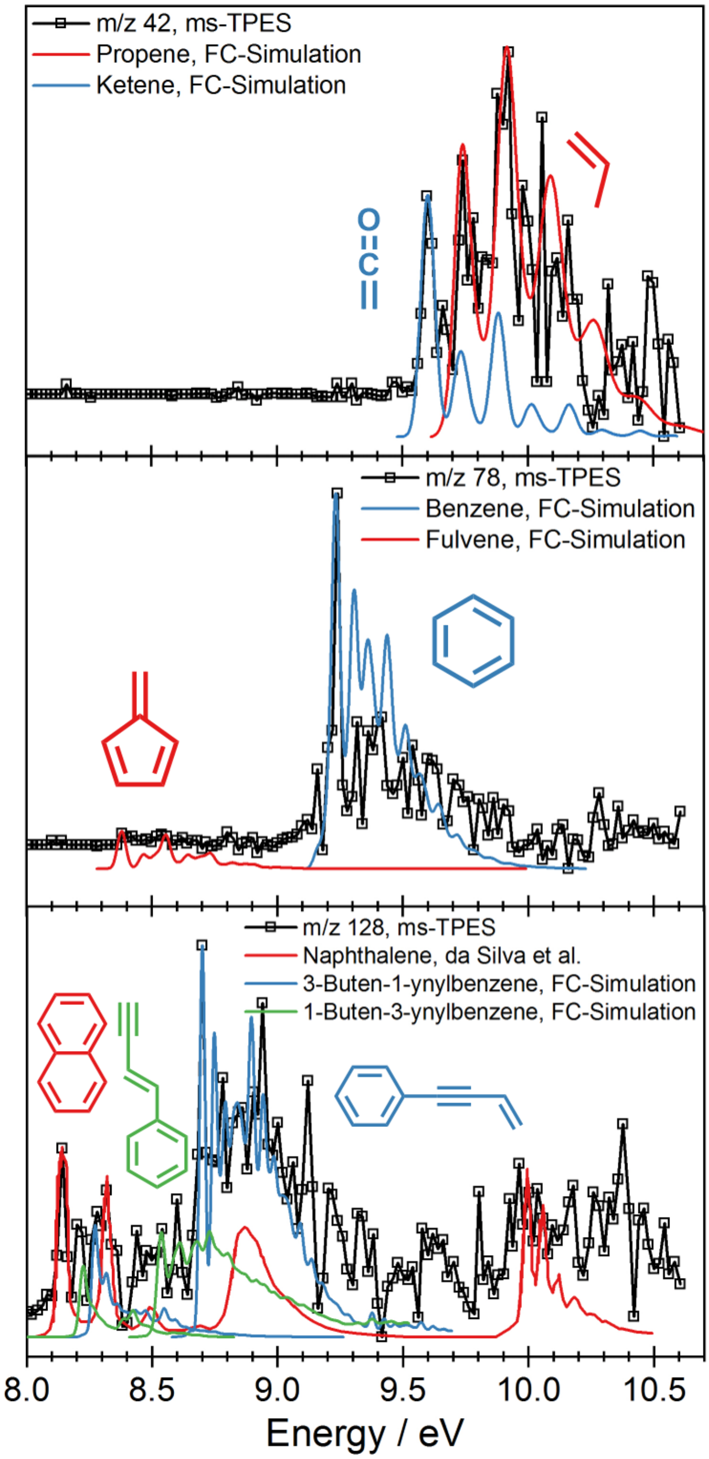

Figure S8. ms-TPE spectra of phenol products along with FC simulations and a reference spectrum for napthalene.[30]

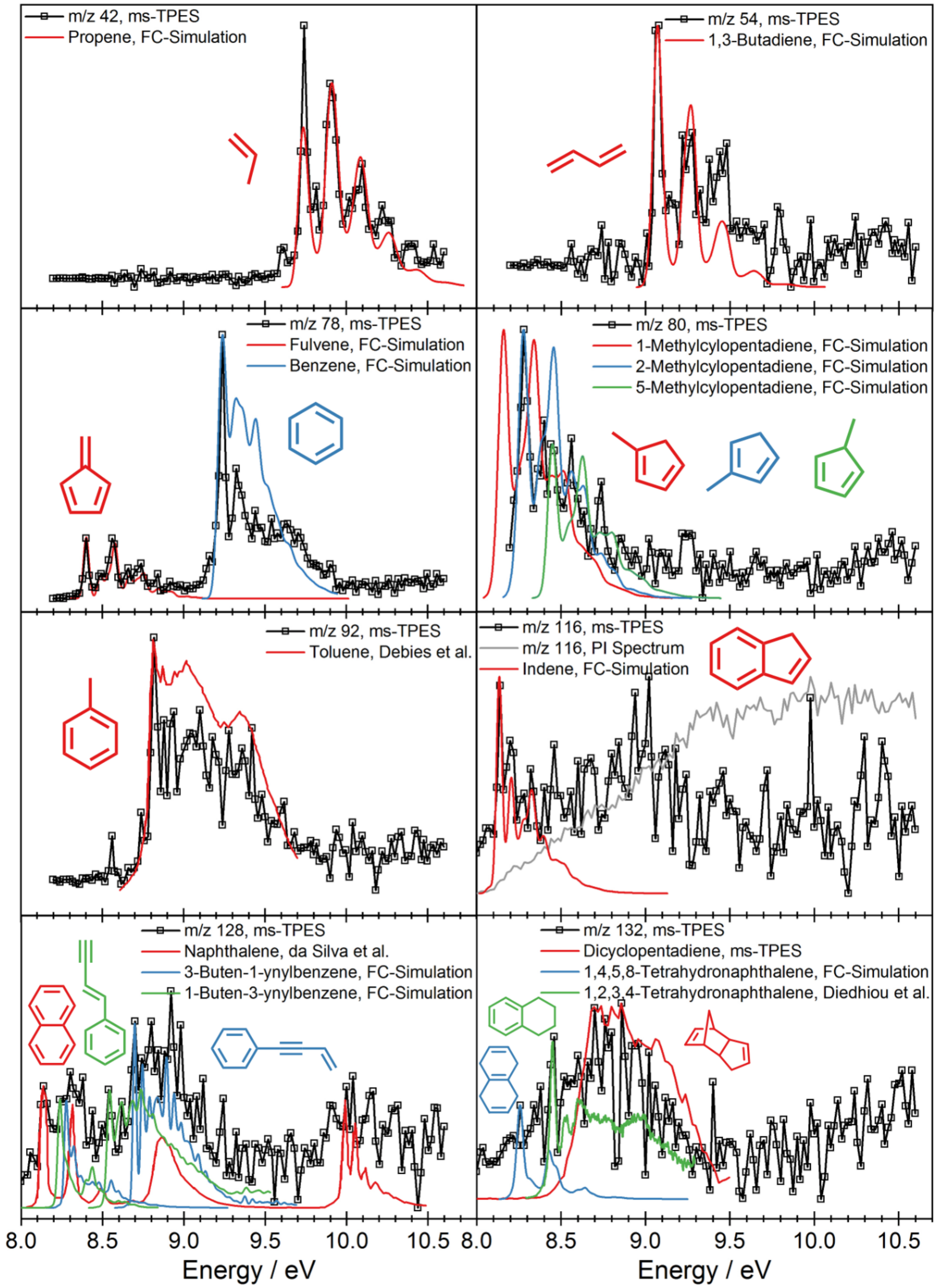

Figure S9. ms-TPE spectra of cyclopentadiene products along with FC simulations and reference spectra.[30-32]

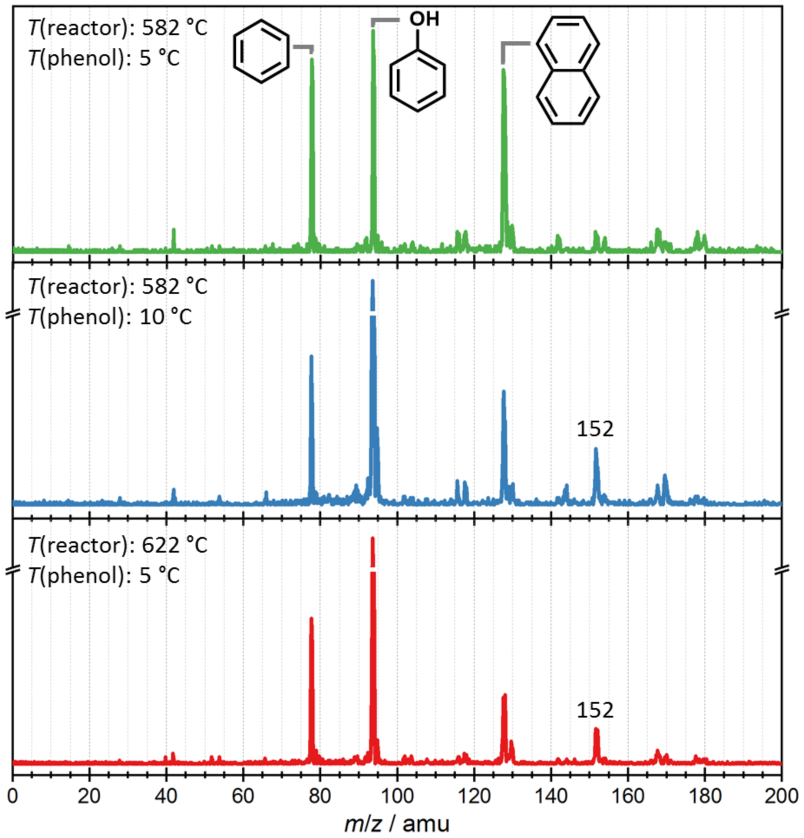

Figure S10. Time-of-flight mass spectra of phenol (*hv* = 10.5 eV) catalytic pyrolysis at different temperatures and sample concentrations controlled by *T*(phenol). Through the investigation of high phenol concentration (blue trace) and reaction temperature (red trace), *m*/*z* 152 was observed and can tentatively be assigned as ethynyl naphthalene or acenaphthylene, which may be produced by the recombination of acetylene with naphthalene according to the hydrogen abstraction–acetylene addition (HACA). Note that acetylene (m/z 26) is not visible at 10.5 eV due to its higher ionization energie of 11.40 eV.

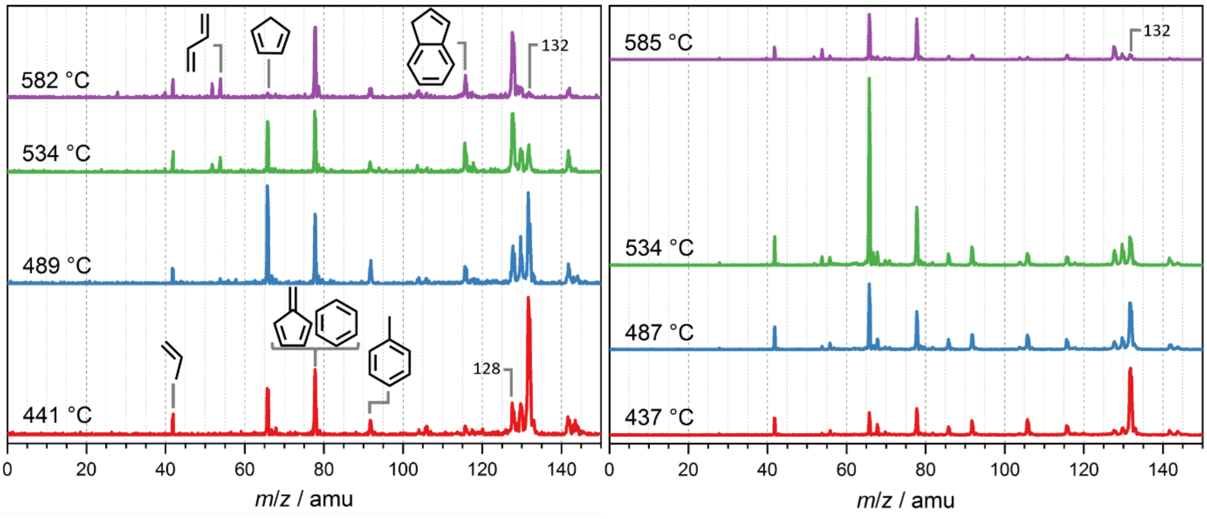

Figure S11. Time-of-flight mass spectra ($h\nu$ = 10.5 eV) obtained upon catalytic pyrolysis of cyclopentadiene (left) and dicyclopentadiene (right). The concentration of cyclopentadiene and dicyclopentadiene is 0.01% and 0.18%, respectively.

REFERENCES

1. G. Zichittella, M. Scharfe, B. Puértolas, V. Paunović, P. Hemberger, A. Bodi, L. Szentmiklósi, N. López and J. Pérez-Ramírez, *Angew. Chem. Int. Ed.*, 2019, **58**, 5877-5881.
2. B. Sztaray, K. Voronova, K. G. Torma, K. J. Covert, A. Bodi, P. Hemberger, T. Gerber and D. L. Osborn, *J. Chem. Phys.*, 2017, **147**, 10.
3. V. N. Emel'yanenko, M. A. Varfolomeev, V. B. Novikov, V. V. Turovtsev and Y. D. Orlov, *J. Chem. Eng. Data*, 2017, **62**, 2413-2422.
4. S. P. Verevkin and S. A. Kozlova, *Thermochim. Acta*, 2008, **471**, 33-42.
5. A. Bodi, M. Johnson, T. Gerber, Z. Gengeliczki, B. Sztaray and T. Baer, *Rev. Sci. Instrum.*, 2009, **80**, 7.
6. R. B. Moffett, *Org. Synth.*, 1952, **32**, 41-44.
7. D. S. Parker, R. I. Kaiser, T. P. Troy and M. Ahmed, *Angew. Chem. Int. Ed.*, 2014, **53**, 7740-7744.
8. V. A. Mozhayskiy and A. I. Krylov, *ezSpectrum*, http://iopenshell.usc.edu/downloads.
9. S. Y. Liang, P. Hemberger, J. Levalois-Grutzmacher, H. Grutzmacher and S. Gaan, *Chem. Eur. J.*, 2017, **23**, 5595-5601.
10. L. A. Curtiss, P. C. Redfern and K. Raghavachari, *J. Chem. Phys.*, 2007, **127**, 124105.
11. T. K. Ormond, P. Hemberger, T. P. Troy, M. Ahmed, J. F. Stanton and G. B. Ellison, *Mol. Phys.*, 2015, **113**, 2350-2358.
12. P. Hemberger, Z. Pan, A. Bodi, J. A. van Bokhoven, T. K. Ormond, G. B. Ellison, N. Genossar and J. H. Baraban, *ChemPhysChem*, 2020, **21**, 2217-2222.
13. G. Bieri, F. Burger, E. Heilbronner and J. P. Maier, *Helv. Chim. Acta*, 1977, **60**, 2213-2233.
14. J. Vogt, A. D. Williamson and J. L. Beauchamp, *J. Am. Chem. Soc.*, 1978, **100**, 3478–3483.
15. J. H. D. Eland, *Int. J. Mass. Spec.*, 1969, **2**, 471-484.
16. D. Chadwick, D. C. Frost, A. Katrib, C. A. McDowell and R. A. N. McLean, *Can. J. Chem.*, 1972, **50**, 2642-2651.
17. T. Koenig, M. Smith and W. Snell, *J. Am. Chem. Soc.*, 1977, **99**, 6663-6667.
18. S. Cradock, E. A. V. Ebsworth, H. Moretto and D. W. H. Rankin, *J. Chem. Soc., Dalton Trans.*, 1975, **5**, 390-392.
19. G. Hentrich, E. Gunkel and M. Klessinger, *J. Mol. Struct.*, 1974, **21**, 231-244.
20. L. Weiler, D. Chadwick and D. C. Frost, *J. Am. Chem. Soc.*, 1971, **93**, 4320-4321.
21. J. C. Traeger and R. G. McLoughlin, *Int. J. Mass. Spec.*, 1978, **27**, 319-333.
22. H. Bock, T. Hirabayashi and S. Mohmand, *Chem. Ber.*, 1981, **114**, 2595-2608.
23. M. J. S. Dewar and S. D. Worley, *J. Chem. Phys.* , 1969, **50**, 654-667.
24. T. Kobayashi, *J. Electron Spectrosc. Relat. Phenom.*, 1975, **7**, 349-353.
25. M. J. S. Dewar, E. Haselbach and S. D. Worley, *Proc. Roy. Soc. Loud. A*, 1970, **315**, 431-442.
26. W. Schäfer, A. Schweig, H. Vermeer, F. Bickelhaupt and H. De Graaf, *J. Electron Spectrosc. Relat. Phenom.*, 1975, **6**, 91-98.
27. F. Brogli, E. Giovannini, E. Heilbronner and R. Schurter, *Chem. Ber.*, 1973, **106**, 961-969.
28. P. Bischof, J. A. Hashmall, E. Heilbronner and V. Hornung, *Tetrahedron Lett.*, 1970, **11**, 1033-1036.
29. A. D. Baker, D. Betteridge, N. R. Kemp and R. E. Kirby, *Anal. Chem.*, 1970, **42**, 1064-1073.
30. D. A. da Silva Filho, R. Friedlein, V. Coropceanu, G. Ohrwall, W. Osikowicz, C. Suess, S. L. Sorensen, S. Svensson, W. R. Salaneck and J. L. Bredas, *Chem. Commun.*, 2004, DOI: 10.1039/b403828b, 1702-1703.
31. M. Diedhiou, B. J. West, J. Bouwman and P. M. Mayer, *J. Phys. Chem. A*, 2019, **123**, 10885-10892.
32. T. P. Debies and J. W. Rabalais, *J. Electron Spectrosc. Relat. Phenom.*, 1972, **1**, 355-370.
